# Dual and spatially resolved drought responses in the Arabidopsis leaf mesophyll revealed by single‐cell transcriptomics

**DOI:** 10.1111/nph.20446

**Published:** 2025-03-03

**Authors:** Rubén Tenorio Berrío, Eline Verhelst, Thomas Eekhout, Carolin Grones, Lieven De Veylder, Bert De Rybel, Marieke Dubois

**Affiliations:** ^1^ Department of Plant Biotechnology and Bioinformatics Ghent University Ghent 9052 Belgium; ^2^ Center for Plant Systems Biology, VIB Ghent 9052 Belgium; ^3^ Single Cell Core Facility, VIB Ghent 9052 Belgium

**Keywords:** *Arabidopsis thaliana*, drought, leaf, mesophyll, single‐cell RNA sequencing, transcriptome changes

## Abstract

Drought stress imposes severe challenges on agriculture by impacting crop performance. Understanding drought responses in plants at a cellular level is a crucial first step toward engineering improved drought resilience. However, the molecular responses to drought are complex as they depend on multiple factors, including the severity of drought, the profiled organ, its developmental stage or even the cell types therein. Thus, deciphering the transcriptional responses to drought is especially challenging. In this study, we investigated tissue‐specific responses to mild drought (MD) in young *Arabidopsis thaliana* (Arabidopsis) leaves using single‐cell RNA sequencing (scRNA‐seq). To preserve transcriptional integrity during cell isolation, we inhibited RNA synthesis using the transcription inhibitor actinomycin D, and demonstrated the benefits of transcriptome fixation for studying mild stress responses at a single‐cell level. We present a curated and validated single‐cell atlas, comprising 50 797 high‐quality cells from almost all known cell types present in the leaf. All cell type annotations were validated with a new library of reporter lines. The curated data are available to the broad community in an intuitive tool and a browsable single‐cell atlas (http://www.single‐cell.be/plant/leaf‐drought). We show that the mesophyll contains two spatially separated cell populations with distinct responses to drought: one enriched in canonical abscisic acid‐related drought‐responsive genes, and another one enriched in genes involved in iron starvation responses. Our study thus reveals a dual adaptive mechanism of the leaf mesophyll in response to MD and provides a valuable resource for future research on stress responses.

**Table:** 

	Content	
	Summary	840
I.	Introduction	840
II.	Materials and Methods	841
III.	Results	844
IV.	Discussion	852
	Acknowledgements	854
	References	855

## I. Introduction

Drought is one of the most challenging stresses for agriculture, imposing significant limitations on crop production and yield (Boyer, 1982). Even when moderate, the decrease in water content initiates stress signaling cascades triggering adaptive mechanisms in root and shoot. The response and severity of the symptoms vary widely depending on the stress level and the developmental stage at which the stress occurs (Fuad‐Hassan *et al*., 2008; Araus *et al*., 2012; Bledsoe *et al*., 2017; Verbraeken *et al*., 2021). Vegetative growth is a particularly vulnerable stage at which drought can severely affect plant performance and the timing of transition to the reproductive stage. To cope with drought, plants activate adaptive mechanisms, including changes in root architecture and an overall shoot biomass reduction. Limiting shoot growth reduces the evaporative surface of the plants and safeguards energy resources, which can be invested in drought resilience mechanisms in case of life‐threatening drought levels (Claeys & Inzé, 2013). As such, understanding plant responses to reduced water availability is essential to develop strategies that enhance plant performance and resilience under drought stress (Muller *et al*., 2011; Claeys & Inzé, 2013; Martignago *et al*., 2019; Dubois & Inzé, 2020; Simmons *et al*., 2021).

In the shoot, adaptive responses vary depending on the tissue, and even within the different cell types of the same tissue. In the leaf epidermis, stomatal closure is one of the first responses to maintain leaf water potential at the onset of drought (Bertolino *et al*., 2019; Laxa *et al*., 2019). In parallel, epidermal cell divisions and expansion of differentiating cells are limited to constrain leaf growth (Granier *et al*., 2006; Baerenfaller *et al*., 2012; Clauw *et al*., 2016; Dubois *et al*., 2017; Chen *et al*., 2021). These cellular growth regulation mechanisms occur before growth reduction is observed at the organ level, within multiple days upon stress onset (Dubois *et al*., 2017). Also in the inner leaf tissues, environmental stress responses are not homogenous: for example, vascular and mesophyll cells display different responses to abiotic stressors, such as UV stress (Berkowitz *et al*., 2021). In our previous study, we observed a trend for mesophyll cells to be strongly responsive to drought at the transcriptome level, but could not study specific responses in depth (Tenorio Berrio *et al*., 2022).

In the last decade, plant responses to moderate drought levels have been extensively studied using bulk transcriptome profiling (Harb *et al*., 2010; Wilkins *et al*., 2010; Baerenfaller *et al*., 2012; Ma *et al*., 2014; Clauw *et al*., 2016; Dubois *et al*., 2017), providing a broad overview of gene expression changes, yet possibly overlooking the tissue‐specific responses. Single‐cell and single‐nucleus RNA sequencing (sc/snRNA‐seq) technologies provide powerful tools to dissect the unique transcriptomic signature of individual cell types or cell populations during plant development or during stress responses (recently reviewed in Nolan & Shahan, 2023; Zhu *et al*., 2023b; Grones *et al*., 2024; Tenorio Berrio & Dubois, 2024). Both scRNA‐seq and snRNA‐seq are routinely used methods, each with their own assets and drawbacks that are of importance in the study of stress responses. Generally, snRNA‐seq is preferred because it allows the capture of dynamic stress‐responsive gene expression changes occurring in the nucleus and does not require enzymatic cell wall digestion, a process for which some organs or species are particularly recalcitrant (Grones *et al*., 2024). However, snRNA‐seq also involves some drawbacks compared with scRNA‐seq, being, for instance, a lower transcriptome coverage (Ding *et al*., 2020; Tian *et al*., 2020; Farmer *et al*., 2021; Kao *et al*., 2021; Conde *et al*., 2022; Neumann *et al*., 2022; Wang *et al*., 2023; Zheng *et al*., 2023). Additionally, snRNA‐seq does not capture cytoplasmic and chloroplastic RNA, a potential contributor to the leaf responses to stresses that impact chloroplastic processes. By contrast, scRNA‐seq offers higher transcriptome coverage but requires enzymatic digestion of cell walls to isolate single cells in the form of protoplasts (Birnbaum *et al*., 2005; Wang *et al*., 2021; Z. Liu *et al*., 2023). Cell wall digestion affects gene expression, and can thereby introduce artifacts and biases in transcriptome analyses that might hinder the interpretation of mild responses upon treatments (Birnbaum *et al*., 2003; Wang *et al*., 2021). Fixing the transcriptional state of the samples before cell wall digestion has been proposed to overcome this issue (Procko *et al*., 2022; Grones *et al*., 2024; Wu *et al*., 2024). While transcriptome fixation is often used in scRNA‐seq studies in the animal field (Attar *et al*., 2018; Wohnhaas *et al*., 2019; Sunkara *et al*., 2021; Pennitz *et al*., 2023), the possibility of applying it to plant samples to counteract the transcriptional response to cell wall digestion and thereby improve the understanding of plant single‐cell responses to mild stress conditions remains unexplored.

In this study, using scRNA‐seq, we dissected the tissue‐specific responses to mild drought (MD) in young Arabidopsis leaves at a stage at which cellular growth restriction mechanisms are expected to be activated, while no indicators of severe drought responses, such as wilting or anthocyanin accumulation, are visible yet. To preserve the drought‐responsive transcriptional changes during cell wall digestion, we fixed the transcriptional state of the samples. For this, the samples of drought‐treated plants and well‐watered (WW) controls were treated with actinomycin D (ActD), an anticancer drug blocking RNA synthesis by intercalating into DNA (Perry & Kelley, 1970; Sobell, 1985) and as such preventing additional transcriptional changes during cell isolation. This treatment improved the study of responses to MD, revealing responses that were largely masked or perturbed by cell isolation without transcriptome fixation. Our approach revealed two distinct drought‐responsive mesophyll cell populations, one enriched in classical, abscisic acid (ABA)‐related drought‐responsive genes, and another one associated with iron starvation. Using reporter lines and RNA fluorescence *in situ* hybridization (HCR‐FISH), we located these two cell populations in two spatially separated regions of a drought‐stressed young leaf, paving the way toward a better understanding of the drought‐adaptive mechanisms in Arabidopsis leaves.

## II. Materials and Methods

### 1. Plant material

*Arabidopsis thaliana* (L.) Heynh Columbia‐0 wild‐type plants were used for all experiments. Reporter lines were generated by amplifying the 2‐kb genomic sequence upstream of the start codon (or up to the closest gene) for 25 selected marker genes (primers in Supporting Information Table S1). The PCR fragment was cloned into pGGA000 via Gibson cloning, as described previously (Decaestecker *et al*., 2019). The following modules were assembled in pFASTRK‐AG via a BsaI‐mediated Golden Gate reaction: promoter in pGGA000, nls‐GFP (without STOP‐codon) in pGGB000, β‐glucuronidase (GUS) in pGGC000 and 35S‐terminator in pGGF000 with linkers where appropriate. After transformation, primary transformants were selected via the FAST system and homozygous lines were obtained. The library of new leaf‐tissue reporter lines is freely available upon request. A similar strategy was used for the generation of the COR15A reporter line. For the double reporter line, the pCOR15A::nls‐mCherry line was crossed with pbHLH100::nls‐GFP described in Radoeva *et al*. (2019) and F1 progeny seeds were used.

### 2. Growth conditions

Arabidopsis was grown under a long‐day regime (16 h : 8 h, light : dark, intensity 110–120 μmol m^−2^ s^−1^) at 21°C, on the weighing, imaging and watering automated machine (WIWAM, http://www.wiwam.be) as described in our previous work (Tenorio Berrio *et al*., 2022). Briefly, the seeds were sown in 85 ± 0.5 g of Saniflor compost (Van Isreal N.V., Geraardsbergen, Belgium) with an average absolute water content of 70%. Five seedlings were grown per pot in order to obtain 80 seedlings per sample (per replicate, per condition). Plants were watered daily until 8 d after stratification (DAS) with a WW regime of soil relative water content 69% (2.2 g_water_/g_soil_). At 9 DAS, half of the pots (randomized) were not watered until harvest (14 DAS, MD), while the other half kept the WW regime. At this stage, no indicators of severe drought could be observed yet. For single‐cell and bulk transcriptomics, two experiments were performed fully independently from each other (*c*. 6 months between).

### 3. Sample preparation for bulk and scRNA‐seq

For the bulk RNA‐seq of undigested leaves, six third true leaves were pooled per condition, per biological replicate, immediately frozen in liquid nitrogen and homogenized with the Retsch MM200. All scRNA‐seq and bulk samples of digested leaves were processed as follows. The cell wall digestion protocol was adapted from Ryu *et al*. (2019). Briefly, the third leaf of 80 WW and 80 drought‐stressed plants per sample were harvested and chopped in 1 ml enzyme solution (0.4 M mannitol (M1902; Sigma), 10 mM CaCl_2_ (C5670; Sigma), 20 mM MES (M1503; Duchefa, Haarlem, The Netherlands), 20 mM KCl (1.049.360.500; Merck, Darmstadt, Germany), 2% (wt/vol, 5.7 pH) cellulase R10 (Yakult Pharmaceutica – Onozuka, Tokyo, Japan) and 0.5% (wt/vol) macero enzyme (R10, L0021; Yakult, Tokyo, Japan)). Chopped samples were transferred to a 70‐μm cell strainer (model Corning, CLS431751; Merck) placed within a 6‐well plate, in a total volume of 5 ml of the enzyme solution. For the Fixed samples, we also harvested the third leaf of 80 WW and 80 drought‐stressed plants per sample and added 50 μM of ActD (A9415; Sigma) to the cell wall digestion solution immediately after chopping. Samples were incubated, constantly shaking for 75 min at room temperature. After a 7 min centrifugation at 200 ***g***, the pellet was resuspended in 255 μl washing solution (0.4 M mannitol, 10 mM CaCl_2_, 20 mM MES and 20 mM KCl). Alive cells were enriched using magnetic levitation (LeviCell from Levitas Bio) by adding 45 μl of Levitation Agent (ref. 1003001; Fisher 12‐852‐112) to the cell suspension, followed by the separation of alive cells from dead cells and debris for 5 min. Subsequently, the purified cell suspension was retrieved from the LeviCell cartridge S2.3 (10‐pk, Ref. 1002010; Fisher 12‐852‐110) and counted using a Fast‐Read® 102 counting slide. A total of 25 000 cells per sample were loaded in the Rhapsody (BD) or kept for bulk RNA‐seq.

### 4. RNA extraction, library preparation and bulk RNA‐seq analysis

Total RNA was isolated using the ReliaPrep RNA Extraction Kit (Promega, Madison, WI, USA) according to the manufacturer's instructions. All samples were further processed by BGI Tech Solutions (Warszawa, Poland). The library was sequenced on the DNBseq platform, and *c*. 30 M 150‐bp pair‐end reads were aimed for during sequencing. After initial filtering by BGI, the number of reads retrieved for each sample was *c*. 20 M. Subsequently, the data were processed using Galaxy. Reads were mapped on the Arabidopsis genome (TAIR 10) using the RNA‐seq aligner tool STAR (Dobin *et al*., 2013). Correlation analysis between the different samples was performed by averaging the normalized expression value of each gene over the replicates and subsequently comparing the log_2_ of each average expression between two samples, or by comparing the log_2_ fold change (FC) of MD vs WW conditions between two samples. Statistical analysis was performed using the deseq2 package v.1.42.1 (Anders & Huber, 2010). Differential expression analysis between two conditions/groups was performed using the Wald test. For more conditions/groups, the likelihood ratio test (LRT) was used. The false discovery rate (FDR) was controlled by adjusting *P*‐values using the Benjamini and Hochberg's approach (Anders & Huber, 2010). Differentially expressed genes (DEGs) were defined as genes with a FDR < 0.05. The DEG distribution was mapped out using plots, which were generated in R using the enhancedvolcano package v.1.20.0 (Blighe & Lewis, 2019). The DEGs identified by each LRT were analyzed using the ‘degreport’ package v.1.38.5 to identify patterns and generate clusters (Pantano, 2024). Scaling the gene expression was done using the *Z*‐score, representing the number of SD below or above the observation mean. The *Z*‐score was calculated by applying the function scale() and relies on the formula *Z* = (*X*–*μ*)/*σ*, where *X* is the expression value of a gene, *μ* is the mean expression, and *σ* is the SD. The *Z*‐score normalizes expression values for each gene across samples, allowing for comparison of patterns regardless of their absolute expression levels, even if those are vastly different. Cluster calculation was based on Kendall correlation of the original (not scaled) values; scaled values were only used for plotting and visualization. A list of top cell wall digestion‐responsive genes was obtained from the top 250 genes in Cluster #1 of the cell wall digestion response, after removal of those cell wall digestion‐responsive DEGs by drought (Table S2). Upset plots were generated to visualize the intersections in the responses to drought in the different samples using the ‘upsetr’ package v.1.4.0.

### 5. GO enrichment analysis

Gene Ontology (GO) enrichment analysis was performed using the ‘clusterprofiler’ package v.4.13.0 (Yu *et al*., 2012). Analysis was performed with the Benjamini and Hochberg's adjustment method, with a *q* value cutoff of 0.4, and the results were simplified using the simplify() function. A less stringent threshold of 0.8 *P*‐value was used for the tissue. The pheatmap package v.1.0.12 was used for visualization (Kolde, 2019).

### 6. scRNA‐seq sample preparation, library construction and sequencing

For each sample, 25 000 cells were loaded on separate lanes of a BD HT Cartridge. Reverse transcription, cDNA amplification and library construction were performed following the manufacturer's instructions (23‐24 117(02)). Libraries were sequenced by VIB Nucleomics Core (Leuven, Belgium) on a NovaSeq 6000 flow cell (Illumina, San Diego, CA, USA).

### 7. Raw data processing and scRNA‐seq analysis

The BD Rhapsody Sequence Analysis Pipeline (BD; v.2.0) was used to map the fastq files to the *Arabidopsis* reference genome (tair10). We utilized seurat (v.5.0.1) in R v.4.16, to process and filter the expression matrix (Hao *et al*., 2024). Information and metadata parameters of the dataset are summarized in Table S3. For each sample, low‐abundance genes (all genes that were expressed in < 5 cells) were removed. Cells expressing < 1000 genes or with < 1250 unique molecular identifiers (UMIs) were filtered out. Normalization, detection of highly variable genes, scaling, clustering and dimensionality reduction were performed using Seurat. The absence of a strong doublet effect on the clustering was verified as the doublet score calculated with scds, with an average hybrid score lower than 0.25 for all samples (Bais & Kostka, 2020). harmony (v.0.1.0; Korsunsky *et al*., 2019a) was used to integrate the data across repeats. The combined dataset is integrated both across repeat and fixation treatment. Uniform manifold approximation and projection (UMAP) was performed using the top 32 harmony‐adjusted principal components, followed by clustering using a resolution parameter of 0.8. The digestion response score was assigned to cells using an approach similar to the cell cycle scoring from Seurat. Briefly, the CellCycleScoring() function was adapted to calculate one single score per cell (instead of 2 for cell cycle score), using a gene list consisting of the top 250 cell wall digestion‐responsive genes from the bulk RNA‐seq samples, excluding the genes that interacted with the MD treatment. Differential expression analysis was performed by the Wilcoxon rank sum test using the package presto v.1.0.0 (Korsunsky *et al*., 2019b). A threshold of *P*‐value < 0.05, log_2_ FC > 0.75, for genes expressed in at least 5% of the cells in the population where it is induced, was set to calculate tissue‐specific responses to MD, which were then visualized using the chordDiagram() function included in the ‘circlize’ package v.0.4.16. Upset plots were generated to visualize the intersections in the tissue‐specific responses to drought in selected tissues. Upset plots showing drought responses were generated using the input from the pseudobulk analysis (*P*‐value < 0.05; log_2_FC > 0.5) using the ‘upsetr’ package v.1.4.0. Pseudobulk expression profiles were generated by averaging the expression using the function AggregateExpression(). DESEQ2 was used to perform a pseudobulk differential expression analysis across different cell types or samples.

### 8. Motif enrichment analysis using MINI‐EX

For Motif‐Informed Network Inference based on single‐cell Expression data (MINI‐EX) analysis, we reclustered the subset of mesophyll populations, obtaining 10 unsupervised clusters that were further grouped into three clusters (one expressing the canonical drought response genes, one expressing iron deprivation response genes, and the rest of the mesophyll cells). Next, the FindAllMarkers() function from Seurat was used to generate the input of DEGs. This list was further filtered to select those genes expressed in > 10% of the cells, with a log_2_ FC higher than 0.1 and an adjusted *P*‐value lower than 0.05. Subsequently, the MINI‐EX analysis was performed, including first the generation of a *de novo* coexpression network using ‘grnboost’, with the expression matrix of the mesophyll cells of the WW and MD samples, from the Fixed–Digested sample only. For further MINI‐EX analysis, the default ‘expressionFilter’ (10) and ‘topRegulons’ size of 150 were used, and the ‘topMarkers’ parameter was changed to 250 genes.

### 9. GUS staining

Leaves were collected for GUS staining at 14 DAS. GUS staining was performed after fixation in 80% acetone. Leaves were incubated in GUS buffer (1 mM X‐GlcA (C6891; Immunosource, Schilde, Belgium), 0.5% (v/v) Triton X‐100 (X100; Sigma), 1 mM EDTA, pH = 8, 0.5 mM K_3_Fe(CN)_6_ (244 023; Sigma), 0.5 mM K_4_Fe(CN)_6_ (P9387; Sigma) and 500 mM sodium phosphate buffer, pH = 7) at 37°C overnight and cleared for 1–2 d in 100% ethanol. After clearing, samples were embedded overnight in lactic acid (CL00.1367.1000; ChemLab, Zedelgem, Belgium) and imaged with the Leica MZ16 binocular microscope equipped with a TOUPCAM camera.

### 10. Leaf clearing, sectioning and confocal imaging

The leaves were fixed in fixative buffer (4% (w/v) paraformaldehyde (158 127; Sigma Aldrich) in 1× phosphate‐buffered saline (PBS), supplemented with Triton 0.1% (v/v) (X100; Sigma)), applying vacuum (4 × 30 min, mixed in between) to promote infiltration. After removing the fixative and performing three washing steps of 5 min in PBS, samples were incubated in the clearing solution ClearSee‐alpha for 10 d (Kurihara *et al*., 2021). ClearSee‐alpha solution was freshly made by adding sodium sulfite (50 mM (S0505; Sigma)) to the ClearSee (Xylitol (X3375; Sigma), sodium deoxycholate (30 970; Sigma), Urea (CL00.2102.1000; ChemLab)) solution and replaced every 2 or 3 d. For sectioning, samples were embedded in agar (3.5% m/v in PBS) and sectioned in 150 μm sections using the Leica VT1200 S vibratome. Fixed samples were stained overnight with 0.1% (v/v) Renaissance SR2200 in ClearSee at room temperature in the dark for 2 d. Confocal imaging was performed using a Leica SP8X confocal microscope equipped with a Leica SuperK laser and a 40× (HC PL APO CS2, NA = 1.10) water immersion‐corrected objective or a ×10 (HC PL APO ×10/0.40 C92) dry objective.

### 11. Fluorescence *in situ* hybridization

The method described by Huang *et al*. (2023) was used, with the following specifications or modifications. The hybridization probes targeting the *BGLU18* and *TSA1* transcripts were designed by and purchased from Molecular Instruments (lot numbers: RTL889 and RTL890, respectively). HCR™ amplification hairpins labeled with the 488 fluorophore, compatible with Renaissance SR2200, were purchased from the same provider. Samples were fixed in 5 ml FAA (7.44% formaldehyde (47 608; Sigma), 5% glacial acetic acid and 50% ethanol in water) in 6‐well plates by vacuum infiltration for 1 h. Dehydration was performed by 10‐min treatments with 70, 90, and 100% ethanol followed by two 5‐min washing steps with methanol. Rehydration was done by sequential washing with 75, 50 and 25% methanol in DPBS‐T water for 5 min per step (DPBS, D8662; Sigma), before being transferred to DPBS‐T for 5 min. The leaves were subsequently incubated in a 10× dilution of the cell wall digestion stock (Huang *et al*., 2023) for 5 min, washed for 5 min in DPSB‐T, fixed again in FAA for 15 min and washed twice with DPBS‐T for 5 min. A proteinase‐K (19 133; Qiagen, Hilden, Germany) treatment was performed as described previously (Huang *et al*., 2023). Samples were subsequently transferred to FAA for 30 min before being washed twice with DPBS‐T. Next, samples were prehybridized by incubation in 30% probe hybridization buffer for 30 min at 37°C and placed overnight at −20°C. Following this, the samples were transferred to 1.2 ml of 30% probe hybridization buffer containing 9.6 μl of each hybridization probe set (1 μM stock) and incubated overnight at 37°C. Subsequently, samples were washed twice for 30 min in 30% probe wash buffer at 37°C and twice for 10 min in 5× SSCT (UltraPure SSC 20×, 15 557 044; Thermo Fisher, Waltham, MA, USA). Next, samples were preamplified for 30 min in amplification buffer. Hairpin solutions were prepared according to Huang *et al*. (2023), using 3 pmol of hairpin h1 and 3 pmol of hairpin h2. After overnight incubation with hairpins, excess hairpins were washed away by three washes (20 min each) in SSCT (saline sodium citrate buffer with 0.1% Tween‐20) buffer. Finally, the samples were cleared in ClearSee‐alpha as described above.

### 12. Image analysis

Quantification of fluorescence intensity was performed using the fiji–imagej software. In brief, we used the split colors tool on each confocal image. Next, the total intensity signal for each gene was calculated in a selected area.

## III. Results

### 1. Transcriptome fixation using actinomycin D preserves the transcriptional responses to mild drought

**Fig. 1:**
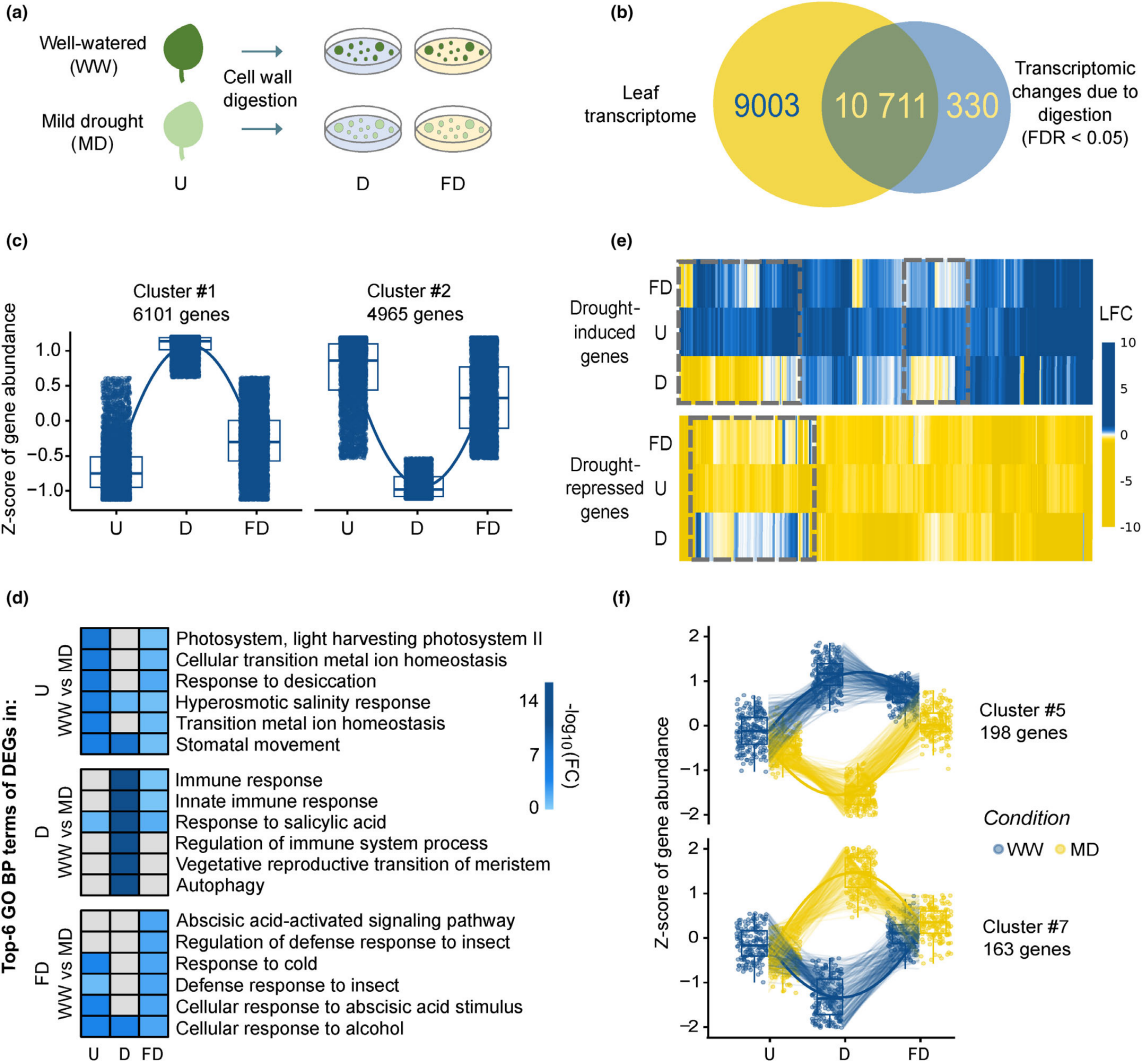
Cell wall digestion responses in the Arabidopsis leaf and interaction with the study of mild drought. (a) Schematic representation of the experimental workflow for bulk and single‐cell RNA sequencing (scRNA‐seq) samples. Bulk RNA‐seq was performed in the undigested leaf (U) sample (*n* = 3) from plants grown under well‐watered (WW) or mild‐drought (MD) conditions, and in samples that underwent cell wall digestion with or without fixation (digested leaf, D and fixed and digested leaf, FD) (*n* = 2). scRNA‐seq was performed with both D and FD samples (*n* = 2). (b) Venn diagram depicting the transcriptomic impact of cell wall digestion (differential expression analysis WW, D vs WW, U, false discovery rate (FDR) < 0.05) compared with the leaf transcriptome (all transcripts present in WW, U). (c) k‐means clustering performed on the differentially expressed genes (DEG) captured upon cell wall digestion. The two most abundant DEG clusters are shown (the rest of the clusters is shown in Supporting Information Fig. S2). For each sample, a boxplot represents the dispersion (25–75% interval) of the *z*‐score of gene abundance, a scaled gene expression value depicting how many SD the expression of each gene deviates from the mean. The horizontal line within the boxplots represents the median. (d) Heatmap depicting the top 6 Gene Ontology (GO) terms (biological processes) enriched in each sample and their level of enrichment in all samples. (e) Heatmap depicting, within the different samples, the behavior of the drought‐responsive genes identified from the undigested leaf samples (701 genes). The upper and lower panels represent the log_2_ fold changes (LFC) of the genes up‐ and downregulated in the U sample, respectively. (f) k‐means clustering performed on the DEGs captured upon interaction analysis between the isolation method and growth condition. The two most abundant DEG clusters are shown (the rest of the clusters are shown in Fig. S6). For each sample, a boxplot represents the dispersion (25–75% interval) of the *z*‐score of gene abundance in each plant growth condition, WW or MD. The horizontal line within the boxplots represents the median.

Because enzymatic digestion during protoplast generation from roots was shown to trigger strong transcriptional responses (Birnbaum *et al*., 2003; Wang *et al*., 2021), we speculated that the transcriptomic impact of cell wall digestion might mask the subtle responses to MD treatment, which were difficult to detect in our previous single‐cell dataset (Tenorio Berrio *et al*., 2022). To circumvent this issue, we explored the use of transcriptome fixation during protoplast generation. Using bulk RNA‐seq, we recorded the transcriptomic changes caused by the enzymatic cell wall digestion during protoplast generation to obtain a set of cell wall digestion‐responsive genes in the actively growing Arabidopsis leaves (third true leaf at 14 d after sowing, at this stage composed of dividing and differentiating cells, from WW conditions only; Fig. 1a). Enzymatic digestion during protoplast generation (for simplicity, the corresponding samples are named ‘Digested’ samples) led to the differential expression of 11 041 genes (including 330 genes exclusively expressed in Digested samples) compared with the Undigested full leaf (further named ‘Undigested’ samples) used as a control (FDR < 0.05; or 6707 genes with Log_2_ FC > 1, FDR < 0.01), representing *c*. 55% of the 19 714 genes captured in the leaf transcriptome of the Undigested samples (Figs 1b, S1A,B; Tables S2, S4). Subsequently, we explored the potential of ActD treatment to block the response to cell wall digestion by comparing the transcriptome of samples fixed during digestion (further named ‘Fixed–Digested’ samples) with that of the Digested and Undigested samples (here as well, only from WW samples). Four distinct gene expression profiles were identified when comparing the cell wall digestion‐responsive genes in all three sample types (Figs 1c, S2A; Table S2). For a minority of the genes (1309 in Cluster 3 and 1412 in Cluster 4), protoplast generation in the presence of ActD triggered expression changes that were more pronounced than without ActD (Fig. S2A). These clusters were slightly biased toward transcripts that are particularly short‐lived (Cluster 3) or stable (Cluster 4), which could explain their altered level when the generation of new transcripts is inhibited by ActD (Table S2; Narsai *et al*., 2007). In addition, GO enrichment analysis of these gene sets suggests that protoplast generation is possibly biased toward photosynthetically active cell types or toward cells with low differentiation levels (Fig. S2B). However, most of these cell wall digestion‐responsive genes displayed an attenuated induction (Cluster 1; 6101 genes) or repression (Cluster 2; 4965 genes) upon cell wall digestion when the samples were fixed, an effect that was not biased by transcript stability (Fig. 1c; Table S2). Overall, the addition of ActD during protoplast generation allowed to retrieve a transcriptome profile that was more closely resembling the profile of an undigested leaf, which was less similar when no transcription inhibitor was added (Fig. S3). Thus, transcriptome fixation during cell wall digestion can mitigate the transcriptomic changes due to protoplast generation in young Arabidopsis leaves.

Next, to evaluate whether transcriptome fixation during the digestion process could preserve the transcriptional changes caused by drought stress, plants were grown on an automated watering platform (Skirycz *et al*., 2011) and were exposed to MD or WW conditions (Fig. 1a). A total of 701 genes (339 down‐ and 362 upregulated; FDR < 0.05) were identified as being differentially expressed in the undigested leaves harvested during drought compared with the undigested leaves from WW plants (Fig. S4A; Table S5). In samples undergoing enzymatic cell wall digestion, either without (Digested) or with fixation (Fixed–Digested), drought affected the expression of 2990 and 286 genes, respectively, compared with the WW samples isolated in the same manner (Fig. S4B,C; Table S5). We observed a significant overlap (20.3× more than expected by chance, Chi‐square *P*‐value = 2.2 × 10^−16^) between the drought‐responsive genes of the Fixed–Digested samples and the Undigested samples, with 43% of DEGs in the Fixed–Digested samples responding to drought in the same way as in Undigested leaves (Fig. S5A,B, Table S5). By contrast, only 5.5% of the drought‐responsive genes in the Digested samples behave similarly in the Undigested leaves (2.5× more than expected by chance, chi‐squared *P*‐value = 2.1 × 10^−11^) (Table S5). In addition, GO analysis of these three sets of drought‐responsive genes showed that biological processes typically associated with drought (e.g. ‘response to desiccation’) were enriched in the Undigested and Fixed–Digested drought‐stressed samples, but not in the Digested drought‐stressed samples (without fixation; Fig. 1d). These results thus suggest that cell wall digestion interferes with the drought response and that, interestingly, transcriptome fixation allows the retention of the drought response upon cell wall digestion of drought‐treated plants.

Finally, we investigated the molecular interaction between drought and cell wall digestion in more detail. We studied the behavior of the 701 drought‐responsive genes from Undigested leaves in the samples that underwent enzymatic cell wall digestion by comparing the FCs upon drought. Surprisingly, Digested samples did not only show different degrees of MD responses, but 195 genes (28% of 701 drought‐responsive genes) even displayed opposite gene expression behaviors in Digested samples compared with the Undigested samples (Fig. 1e). The substantial disruption in the behavior of drought‐responsive genes due to protoplast generation was again mitigated in the Fixed–Digested samples, in which 44 genes (6% of the 701 drought‐responsive genes) showed an opposite gene expression behavior. Further investigation of the statistical interaction between cell wall digestion and drought responses identified up to 1497 significantly interacting genes (*P*_Growth_Condition_ × _Digestion_Treatment_ < 0.05; Figs 1f, S6; Table S6). For most of these genes, fixing the transcriptome during cell wall digestion largely attenuated the interaction effect, without completely blocking it. Taken together, our bulk RNA‐seq experiments suggest that ActD application during protoplast isolation reduces the postharvest transcriptional changes and, thereby, allows the maintenance of the drought‐triggered transcriptional responses in young leaves of drought‐stressed plants.

### 2. An optimized scRNA‐seq atlas for mild‐drought responses in Arabidopsis leaves

**Fig. 2:**
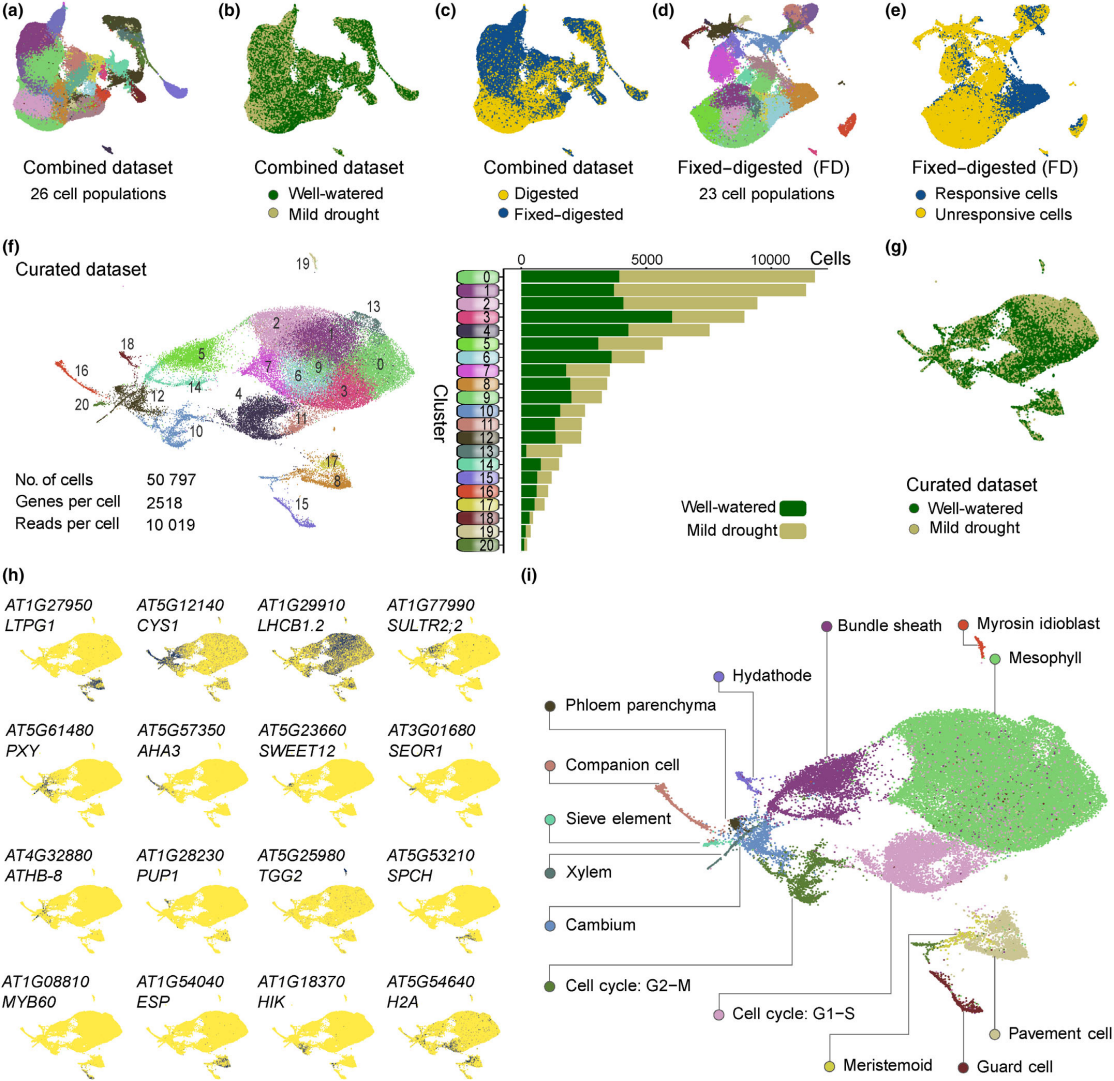
Optimizing and annotating a single‐cell atlas for Arabidopsis leaves upon mild drought. (a–c) Uniform manifold approximation and projection (UMAP) visualization of the combined dataset, containing both Digested and Fixed–Digested samples, following unsupervised clustering (a) or grouped by growth condition (b) or by fixation treatment (c). (d, e) UMAP visualization of the Fixed–Digested samples, grouped by cluster (unsupervised clustering) (d) and by cell wall digestion response score, calculated based on the top 250 cell wall digestion‐responsive genes (e). (f) UMAP visualization of the curated dataset containing cells of the Fixed–Digested samples with a low cell wall digestion response score. Cells are clustered by unsupervised clustering. The right panel represents the normalized number of cells per cluster grouped by growth condition. (g) UMAP visualization of the curated dataset grouped by growth condition. (h) UMAP plots depicting the normalized expression of tissue‐specific marker genes for the epidermis (*LPTG1*), vasculature (*CYS1*), mesophyll (*LHCB1.2*), bundle sheath (*SULTR2;2*), xylem (*PXY*), companion cells (*AHA3*), phloem parenchyma (*SWEET12*), sieve elements (*SEOR1*), cambium (*ATHB‐8*), hydathode (*PUP1*), myrosin idioblast (*TGG2*), meristemoids (*SPCH*), guard cells (*MYB60*), pavement cells (*ESP*) and two populations depicting cell cycle states: G2‐M (*HIK*) or G1‐S (*H2A*). (i) UMAP plot with the annotation of the curated dataset.

To detect the tissue‐specific transcriptional effects of MD in young Arabidopsis leaves, we sampled plants under WW and MD conditions for cell isolation either without (Digested) or with (Fixed–Digested) ActD treatment, in two independent experiments (Fig. 1a). Protoplast suspensions were profiled by scRNA‐seq using the BD Rhapsody microwell‐based system. After quality and doublet rate control (Figs S7, S8), we combined data from all scRNA‐seq samples, retaining a total of 152 793 high‐quality cells with a minimum UMI count of 1250 and a total of 26 150 expressed genes. We subsequently applied batch correction, reducing the batch effect between samples (see Section II) and performed unsupervised clustering (Fig. 2a). From this combined dataset, 85 889 cells were isolated from Digested samples (48 072 WW and 37 817 MD), while 66 904 cells came from Fixed–Digested samples (38 641 WW and 28 263 MD). Although cells from WW and MD samples integrated well throughout the UMAP plot (Fig. 2b), the main source of transcriptional variation was caused by the transcriptome fixation (Figs 2c, S9), supporting our conclusions drawn from the bulk RNA sequencing (Fig. 1c).

Having established the beneficial role of ActD treatment in preserving the drought responses in the leaf, we proceeded with the dataset containing only the Fixed–Digested samples (Fig. 2d). Based on the top 250 cell wall digestion‐responsive genes (calculated comparing the bulk RNA‐seq data from the Digested to the Undigested samples and excluding the genes interacting with the study of MD), we calculated a digestion response score and visualized this score in the different datasets (Fig. S10; Table S2). We noticed that a fraction of the cells in the Fixed–Digested dataset (24%; 16 107 out of 66 904), which were unevenly spread across the different cell populations, seemingly escaped the ActD treatment and still showed a strong cell wall digestion response (Figs 2e, S10). After removing those cells to curate the dataset, the optimized single‐cell atlas of young leaves exposed to MD or WW conditions comprises 50 797 high‐quality cells, expressing a total of 23 717 genes (on average 2518 genes), and grouped by unsupervised clustering into 20 distinct clusters (Figs 2f, S11). Nearly all clusters were composed of a similar proportion of cells from the WW and the MD samples, and no stress‐specific cell clusters were observed (Fig. 2f,g).

### 3. Prediction and experimental validation of cell types

To predict the cluster annotations of the curated dataset obtained from Fixed samples, we analyzed the expression of reported marker genes used for annotation in previous single‐cell studies (Fig. 2h; Kim *et al*., 2021; Tenorio Berrio *et al*., 2022). This allowed us to classify the clusters into 14 distinct cell populations, including 12 cell types, and two cell state populations composed of cells of which the cell type signature is masked by a strong cell cycle state signature (Fig. 2I). The cell type populations were further grouped by main tissue type (mesophyll, epidermis and vasculature), and the cell cycle phase of all populations was predicted (Fig. S12). Following the same method, the datasets of Digested samples and the combined dataset (Digested + Fixed–Digested) were also annotated, allowing the comparison of the cell wall digestion response in the different leaf tissues (Figs S13, S14; Table S7). Although most of the cell wall digestion response was shared across the three main tissues, tissue‐specific responses were also present (Fig. S14).

**Fig. 3:**
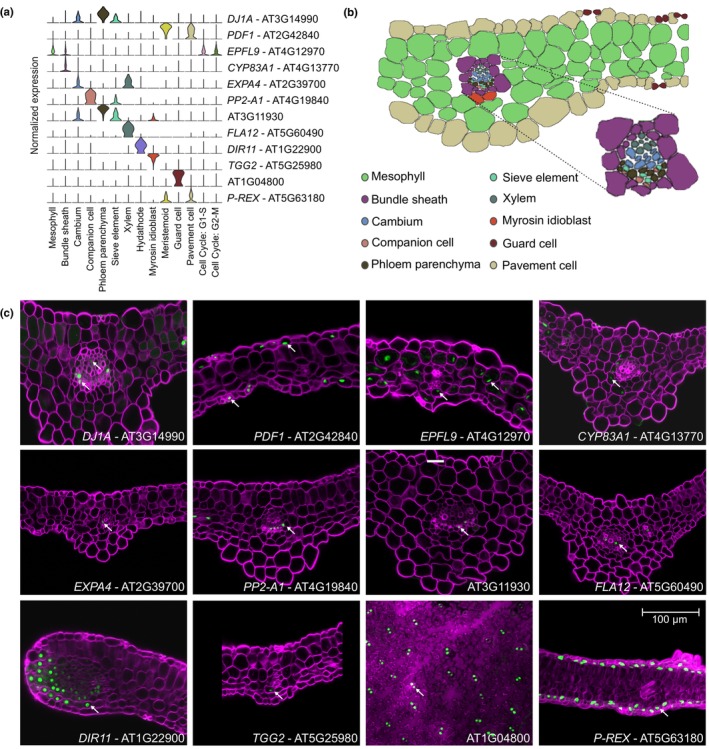
Biological validation of the annotation. (a) Violin plots depicting the tissue‐specific expression of marker genes for the vasculature (*DJ1A*), epidermis (*PDF1*), mesophyll (*EPFL9*), bundle sheath (*CYP83A1*), cambium (*EXPA4*), companion cell (*PP2‐A1*), phloem parenchyma (*AT3G11930*), xylem (*FLA12*), hydathode (*DIR11*), myrosin idioblast (*TGG2*), guard cell (*AT1G04800*) and pavement cell (*P‐REX*), that were selected for the generation of reporter lines. (b) Schematic representation of the section of the Arabidopsis leaf colored by cell type. (c) Confocal imaging of the selected reporter lines expressing the green fluorescent protein (GFP)‐β‐glucuronidase (GUS) reporter construct driven by the promoter of the indicated genes. For all imaging, except for the guard cell reporter line, cross‐sections of cleared Arabidopsis leaves were made to visualize the inner tissues. White arrows point to a GFP‐positive nucleus. The magnification (bar, 100 μm) is the same in all panels.

To validate the predicted annotation of each population, we generated reporter lines for 25 genes with specific expression in most of the predicted cell types, by fusing the promoter region to the nuclear‐localized green fluorescent protein (GFP) and GUS genes (Table 1; Figs 3a, S15, S16). First, we confirmed via GUS staining that the expression pattern of these genes along the leaf corresponded to the expected main tissue (Fig. S16). Next, we selected one representative gene per tissue (Fig. 3a) and analyzed the expression of each of these genes in more detail via confocal microscopy on cross‐sections of leaves (Figs 3b,c, and additional genes in S17). In line with the predicted expression patterns (Fig. 3a – top 3 rows), we found genes expressed in multiple cell types of one of the main tissues (vasculature, epidermis and mesophyll), although their expression levels could differ between the cell types of that tissue. For instance, the vascular marker gene *DJ1A* was expressed along most of the vasculature, with a higher expression in the phloem cells (Fig. 3c). In addition, *EPIDERMAL PATTERNING FACTOR LIKE‐9* (*EPFL9*) and *PROTODERMAL FACTOR1* (*PDF1*) marked the mesophyll and epidermal main tissues, respectively. Due to the photosynthetic nature of the bundle sheath cells, *EPFL9* was also found to be expressed along the bundle sheath (Fig. 3c). Furthermore, we also generated and verified reporter lines for the bundle sheath (*CYTOCHROME P83A1* or *CYP83A1*), for most of the vascular cell types present in the leaf, including the cambium (*EXPANSIN 4* or *EXPA4*), the companion cells of the phloem (*PP2‐A1*), the phloem parenchyma (*AT3G11930*), the xylem (*FASCICLIN‐LIKE ARABINOGALACTAN‐PROTEIN 12* or *FLA12*) and the myrosin idioblast (*TGG2*), and for the epidermal stomata (*AT1G04800*) and pavement cells (*AT5G63180*, here named *PAVEMENT CELL‐RESTRICTED EXPRESSION*; Table 1; Figs 3c, S17). Lastly, the reporter of *DIRIGENT PROTEIN 11* (*DIR11*) marked the hydathodes (Figs 3a,c, S18). Although no reporter line was generated for the sieve elements of the phloem nor for the meristemoids, the identity of the corresponding clusters could be validated based on the expression of the previously reported *SIEVE‐ELEMENT‐OCCLUSION‐RELATED 1* (*SEOR1*) and *SPEECHLESS* (*SPCH*) marker genes, respectively (Fig. 2h; MacAlister *et al*., 2007; Pelissier *et al*., 2008). Taken together, all generated reporter lines confirmed the annotation of the cell clusters of the scRNA‐seq atlas of the young Arabidopsis leaf. Based on the expression patterns of the markers validating each UMAP cluster, we translated the scRNA‐seq data from our optimized dataset into a user‐friendly tool for visualization (http://www.single‐cell.be/plant/leaf‐drought) of any gene expression in a browsable UMAP plot and throughout a schematic Arabidopsis leaf section (Fig. S8).

**Table 1:** Library of tissue‐specific reporter lines.

Cell type	Gene	Name
Epidermis	AT2G42840	*PDF1 – PROTODERMAL FACTOR1*
	AT3G16370	*APG*
Vasculature	AT1G64700	Unknown gene
	AT5G03610	*GGL25*
	AT1G80520	Unknown gene
	AT3G14990	*DJ1A – DJ‐1 HOMOLOG A*
Mesophyll	AT4G26530	*FBA5 – FRUCTOSE‐BISPHOSPHATE ALDOLASE 5*
	AT2G34430	*LHB1B1 – LIGHT‐HARVESTING CHLOROPHYLL‐PROTEIN COMPLEX II SUBUNIT B1*
	AT4G12970	*EPFL9 – EPIDERMAL PATTERNING FACTOR LIKE‐9*
Bundle sheath	AT1G25230	Unknown gene
	AT4G13770	*CYP83A1 – CYTOCHROME P83A1*
Cambium	AT2G39700	*EXPA4 – EXPANSIN 4*
Companion cells	AT4G19840	*PP2A1*
	AT5G18600	*GRXS2 – GLUTAREDOXIN 2*
	AT1G64370	Unknown gene
Phloem parenchyma	AT3G11930	Unknown gene
	AT5G24800	*BZIP9 – BASIC LEUCINE ZIPPER 9*
Xylem	AT3G10080	Unknown gene
	AT5G60490	*FLA12 – FASCICLIN‐LIKE ARABINOGALACTAN‐PROTEIN 12*
Hydathode	AT1G22900	*DIR11 – DIRIGENT PROTEIN 11*
Myrosin idioblast	AT5G26000	*TGG1 – THIOGLUCOSIDE GLUCOHYDROLASE 1*
	AT5G25980	*TGG2 – THIOGLUCOSIDE GLUCOHYDROLASE 2*
	AT3G16400	*NSP1 – NITRILE SPECIFIER PROTEIN 1*
Guard cells	AT1G04800	Unknown gene
Pavement cells	AT5G63180	*PREX – PAVEMENT CELL‐RESTRICTED EXPRESSION/ATPLL15*

### 4. Mesophyll cells display two spatially distinct transcriptional responses to drought

**Fig. 4:**
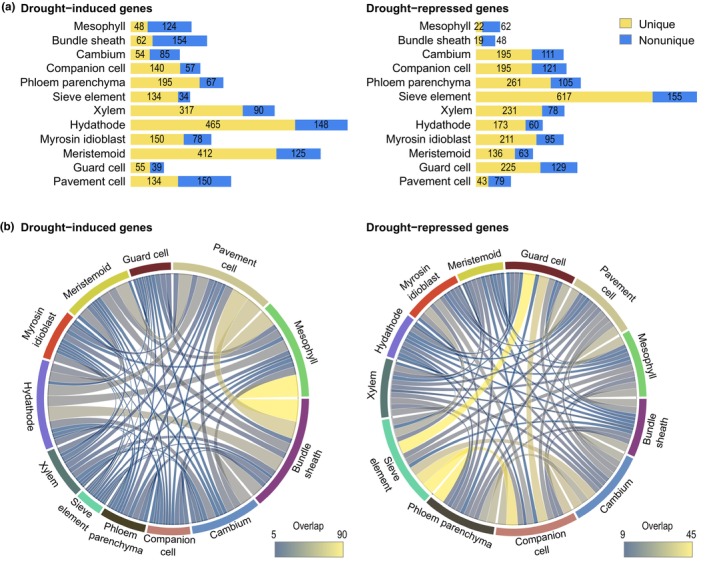
Shared and unique responses to mild drought (MD) in Arabidopsis leaves. (a) Stacked bar plot displaying the number of uniquely and nonuniquely induced and repressed genes for each cell type upon MD, in the left and right panels, respectively. (b) Chord diagram representing the relations between the shared drought‐induced and drought‐repressed responses between the different cell types, in the left and right panels, respectively. Connections are colored by the extent of the shared response, where dark blue and bright yellow connections represent few and large shared responses, respectively. The genes present in each overlap are found in Supporting Information Table S8.

Validating the annotation of each cluster of the single‐cell atlas allowed us to confidently profile the responses to MD per leaf tissue (Table S8). To do so, we made use of the curated scRNA‐seq dataset containing only the Fixed–Digested WW and MD samples (Fig. 2f,g,i). For each cell type, we identified up‐ or downregulated drought‐responsive genes by comparing MD and WW cell transcriptomes per tissue and observed that a fraction of those genes was affected by drought in a tissue‐specific manner (Fig. 4a). Drought responses included GO biological terms associated with, for example, ‘Pectin biosynthesis’ in the xylem, ‘Response to auxin’ in the pavement cells, ‘Response to brassinosteroids’ in the hydathodes, and ‘Response to salicylic acid’ in bundle sheath and guard cells (Table S8). Further analysis of the shared drought‐induced genes highlighted similarities in the response between the mesophyll, the bundle sheath, the pavement cells and the hydathodes (Figs 4b, S19A). On the contrary, the phloem populations, being the companion cells, sieve elements and phloem parenchyma, together with the guard cells, shared a large amount of genes downregulated under drought (Figs 4b, S19B).

**Fig. 5:**
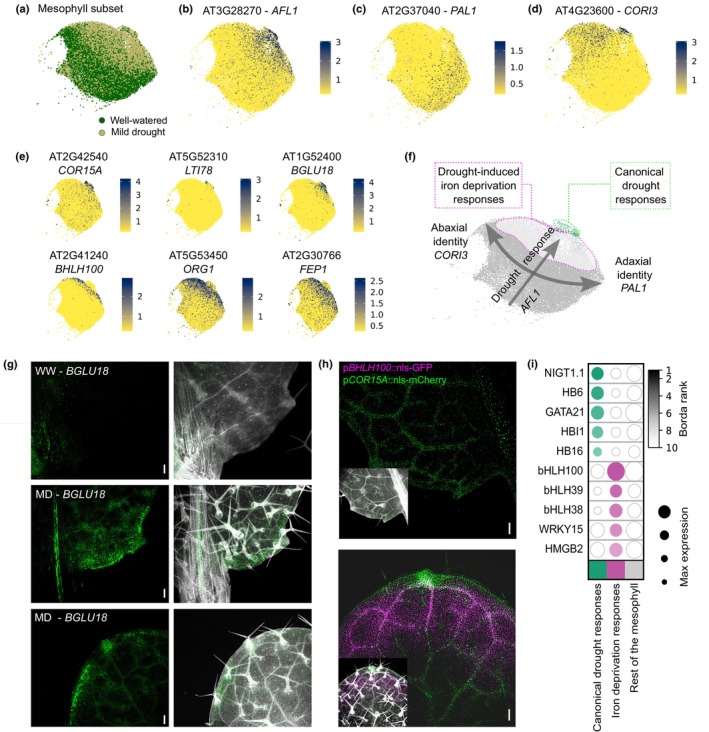
Arabidopsis mesophyll cells display two distinct drought responses. (a) Uniform manifold approximation and projection (UMAP) visualization of the mesophyll subset of cells, grouped by growth condition. (b–d) UMAP plots depicting the normalized expression of markers of drought response (*AFL1*) (b), adaxial polarity (*PAL1*) (c) and abaxial polarity (*CORI3*) (d). (e) UMAP plots depicting the normalized expression of several drought‐responsive genes, expressed either mainly in the tip zone of the mesophyll cluster (*COR15A*, *LTI78* and *BGLU18*), or in a large zone of the mesophyll cluster, but not in the very tip of the cluster (*BHLH100*, *ORG1* and *FEP1*). (f) Schematic representation of the two gradients present in the mesophyll populations: one gradient encompassing the drought response, starting in the zone with mainly well‐watered (WW) sample cells, and ending in the upper right tip of the mesophyll cluster, and another gradient reflecting the leaf polarity. The highlighted parts represent the two drought‐responsive cell populations. (g) Confocal microscopy of whole‐mount fluorescence *in situ* hybridization (HCR‐FISH) targeting the *BGLU18* transcripts, displaying the expression of *BGLU18* upon WW and mild‐drought (MD) conditions. Right images display white‐stained cell walls of the leaf base (top and middle image) and tip (bottom image). Bars, 100 μm. (h) Confocal imaging of green fluorescent protein (GFP) and mCherry expression in a dual *pbHLH100::nls‐GFP* x *pCOR15A::nls‐mCherry* reporter line exposed to MD conditions. Base (left) and tip (right) of the leaf, with the insets displaying white‐stained cell walls of the corresponding zone. Bars, 100 μm. The quantification of the fluorescent signal of the plant lines shown in (h) is shown in Supporting Information Figs S25 and S26. (i) Simplified output of the MINI‐EX analysis run on both drought‐responsive mesophyll populations. The top 5 candidate regulators per population are shown (full output in Fig. S27). MINI‐EX, Motif‐Informed Network Inference based on single‐cell EXpression data.

Although the mesophyll tissue did not appear to be the most responsive cell type based on the number of responsive genes, we studied its drought response in more depth, taking advantage of the high number of cells in this tissue (Fig. 2i). Two interesting observations captured our attention specifically in the mesophyll. First, the mesophyll cluster ‘13’ displayed a visibly higher percentage of drought‐responsive cells after unsupervised clustering compared with any other cluster (Fig. 2f,i). Second, a closer inspection of the UMAP representation of mesophyll cells revealed a clear shift between the mesophyll cells from the WW samples and those from the MD samples, indicating outspoken transcriptome differences (Fig. 5a). For example, the expression of the drought stress marker genes *AT14A‐LIKE1* (*AFL1*), *RESPONSE TO DESICCATION 20* (*RD20*) and *FIBRILLIN1A* (*FIB*) followed this gradient of cells from WW and MD samples (Figs 5b, S20A; Yang *et al*., 2006; Aubert *et al*., 2010; Kumar *et al*., 2015). Consistent with the bulk RNA‐seq results and with our previous study (Tenorio Berrio *et al*., 2022), the drought‐repressed gene *CUPPER SUPEROXIDASE 2* (*CSD2*) was found to be expressed across the opposite gradient (Fig. S20B). These expression patterns support the presence of a gradient in the mesophyll population as displayed in the UMAP plot, with an increasing drought response from the lower left corner (mainly WW cells) toward the upper right corner (mainly MD cells). Perpendicular to this gradient, a second gradient following the abaxial–adaxial leaf polarity axis was observed, reflected by the expression of the adaxial and abaxial mesophyll markers *PHENYLALANINE AMMONIA‐LYASE 1* (*PAL1*) and *CORONATINE INDUCED 3 (CORI3*), respectively (Fig. 5c,d; Procko *et al*., 2022).

Despite some drought‐responsive genes being expressed commonly among the MD mesophyll cells (e.g. *AFL1*), two distinct responses emerged within the group of drought‐responsive mesophyll cells. On the one hand, genes associated with canonical drought response processes like ABA metabolism, for example *BETA GLUCOSIDASE 18* (*BGLU18*), the LEA protein *COLD‐REGULATED 15A* (*COR15A*), *LOW‐TEMPERATURE‐INDUCED 78* (*LTI78*) and multiple other genes (Table S9), were expressed predominantly in the group of cells at the very tip of the drought gradient (Fig. 5e,f, and other examples in Fig. S20C; Hajela *et al*., 1990; Baker *et al*., 1994; Artus *et al*., 1996; Han *et al*., 2020). Notably, a population of cells undergoing such canonical drought response was not detected in the samples without fixation as, for example, the signature gene *BGLU18* was barely expressed in the Digested samples (Fig. S21). On the other hand, drought‐responsive genes primarily associated with responses to iron deprivation or homeostasis, such as *BASIC HELIX–LOOP–HELIX PROTEIN 100* (*bHLH100*), *FE‐UPTAKE‐INDUCING PEPTIDE1* (*FEP1*) and *OBP3‐RESPONSIVE GENE 1* (*ORG1*), were expressed in a large part of the MD mesophyll cells, but almost not in the cells at the very tip of the drought response gradient (Fig. 5e,f, and other examples in Fig. S20D; Table S9; Wang *et al*., 2007; Sivitz *et al*., 2012; Hirayama *et al*., 2018; Kim *et al*., 2019). Consistently, this observation was supported by the enrichment of genes associated with responses to iron ion following an opposite gradient toward the WW‐enriched mesophyll population, including FERRITINs (*FER1* and *FER4*) or *IRON SUPEROXIDASE DISMUTASE 1* (*FSD1*; Fig. S20B; Waters *et al*., 2012). Thus, we observed two gradients within the mesophyll cluster: a leaf polarity gradient and a drought response gradient, with two distinct populations emerging under drought conditions (Fig. 5f).

While the induction of genes like *BGLU18* and *COR15A* under drought was strong and detectable in the bulk RNA‐seq profiling of the full leaf (Table S5), the population of mesophyll cells displaying the canonical drought response was relatively small. To link the unique transcriptional profiles from the scRNA‐seq analysis to a detailed spatiotemporal location within the leaf, we conducted whole‐mount *in situ* hybridizations with probes targeting transcripts of *BGLU18* and *TONSOKU‐ASSOCIATING PROTEIN 1* (*TSA1*), which was also specifically expressed in this canonical drought response population, in WW and drought‐stressed leaves (Figs 5e, S20C). Within the mesophyll, these genes were expressed in small patches of cells situated at the margin of leaves from drought‐stressed plants, but not in WW samples (Figs 5g, S22). This was confirmed through cross‐sections of the leaves (Fig. S22). We also observed additional expression in the phloem region of the midvein upon MD (Fig. S22). In the single‐cell dataset, these genes were expressed upon drought in a very small vasculature population, of which the precise identity remains to be confirmed (Fig. S23).

Next, we investigated whether the population of MD mesophyll cells enriched for iron starvation responses and the small cell population with the canonical drought response reflect two spatially separated mesophyll cell populations within a single leaf. We crossed two reporter lines to simultaneously visualize the expression of *bHLH100* and *COR15A*, two genes rarely coexpressed based on the scRNA‐seq results (Fig. S24). *bHLH100* was highly expressed in the vasculature and mesophyll of the leaves, although only in a region toward the tip of the leaf (Fig. 5h). Conversely, *COR15A* was expressed in mesophyll cells around the vasculature and at the margin of the leaf, aligning with the expression pattern of *BGLU18* (Fig. 5h). Although the fluorescent reporters were also detectable in leaves of WW plants, the signal was increased in drought‐stressed plants (Figs S25, S26). In summary, the spatiotemporal resolution of the scRNA‐seq dataset allowed us to identify two distinct drought‐responsive mesophyll populations in the leaf: one at the margin and around the vasculature and another encompassing the rest of the mesophyll cells. Finally, to study in more detail the regulatory mechanisms that act in both drought‐responsive populations, we performed MINI‐EX analysis on both subclusters (Ferrari *et al*., 2022; Figs 5i, S27; Table S9). This analysis revealed that the genes expressed in the canonical drought response population are enriched for binding sites of the ABA‐responsive HOMEOBOX 6 (HB6), MYB73 and HOMOLOG OF BEE2 INTERACTING WITH IBH 1 (HBI1) transcription factors. Instead, the binding motifs for bHLH100, bHLH39 and bHLH38 were strongly overrepresented in the iron deprivation response population (Figs 5i, S27; Table S9), suggesting that they possibly regulate the responses in this population. Although this observation mainly serves as a starting point for further functional studies on the role of these two cell populations during drought, these data reveal that the seemingly homogeneous mesophyll cells display a dual, early transcriptional response to cope with MD.

## IV. Discussion

In this study, we present a curated, comprehensive scRNA‐seq atlas of young Arabidopsis leaves. Our atlas encompasses the transcriptome of leaves from WW and drought‐stressed seedlings, capturing the vast majority of cell types present in the true leaf. However, some cell types could not be detected in our dataset and are also absent in previous leaf scRNA‐seq atlases, probably due to technical constraints (Kim *et al*., 2021; Lopez‐Anido *et al*., 2021; Procko *et al*., 2022; Tenorio Berrio *et al*., 2022; Zhu *et al*., 2023a). For example, trichomes could not be isolated, neither through scRNA‐seq nor through snRNA‐seq, possibly due to their low number or their large cell and nucleus size, resulting from high ploidy levels (Hulskamp *et al*., 1994; Churchman *et al*., 2006; Delannoy *et al*., 2023). Similarly, the very large glucosinolate‐accumulating S‐cells undergo programmed cell death at an early stage and lack a known transcriptional signature, explaining why they are also unidentified (Koroleva *et al*., 2010; Kim *et al*., 2021; Procko *et al*., 2022; Tenorio Berrio *et al*., 2022; Delannoy *et al*., 2023; Maeda *et al*., 2023; Zhu *et al*., 2023a). Finally, we also lacked a population of large differentiated phloem cells expressing *BGLU18*, possibly because those cells are also recalcitrant to cell wall digestion. In total, 12 distinct cell types and two populations of cells clustered by their cell cycle state were identified. Importantly, the annotation was further validated by reporter lines expressing *GFP‐GUS* in each of these tissues of the young leaf. This atlas is now available as a browsable tool, along with a gene expression visualization tool in a schematic representation of the leaf (http://www.single‐cell.be/plant/leaf‐drought).

Furthermore, our study explores the use of transcriptome fixation before scRNA‐seq to preserve the transcriptomic impact of a subtle treatment, such as MD. Although widely applied in the animal field, tissue fixation in the plant scRNA‐seq field is limited but holds great potential for limiting the impact of cell wall digestion on the plant's transcriptome (Alles *et al*., 2017; Procko *et al*., 2022; Grones *et al*., 2024). Our bulk RNA‐seq on Digested samples (from WW conditions) revealed that more than half of the Arabidopsis leaf transcriptome is affected by cell wall digestion. However, the strong cell wall digestion response did not affect cluster annotation as the expression of the tissue‐specific marker genes was maintained (Fig. S28). The massive transcriptomic changes that we observed are in line with a previous study that found *c*. 80% of the leaf transcriptome responding to overnight cell wall digestion (using the same statistical threshold as our study, FDR < 0.05; M. Xu *et al*., 2021). Thus, transcriptomic changes due to cell wall digestion in the leaf appear to be larger than in root tissues (Birnbaum *et al*., 2005; Chupeau *et al*., 2013). However, whether the leaf is more prone to digestion‐triggered transcriptome reshuffling than roots, or whether other technical differences between these four studies could explain the differences between root and shoot, is not clear. In our study, although the cell wall digestion response was alleviated by transcriptome fixation, it was not completely inhibited. In fact, in the bulk RNA‐seq analysis compared with the undigested leaf, most of the digestion‐responsive genes were still deregulated in the Fixed‐Digested samples. The scRNA‐seq analysis of these samples suggested that there was no basal, general cell wall digestion response but that, instead, differences in the digestion response occurred between distinct cells. However, whether these differences are due to distinct effectiveness of fixation, or to differences in responsiveness to the cell extraction procedure, remains unknown. We speculate that this could be due to some cells being exposed longer to the digestion buffer before the fixative is added. Particularly relevant for our study was the added value of transcriptome fixation to preserve the leaf response to MD, which appeared to be largely interacting with responses induced by the cell isolation procedure. This interaction could be caused, for example, by overlapping transcriptome changes or by drought‐regulating proteins of which the synthesis is altered by ActD. Because of the extent of the drought and cell isolation interaction, it would be inappropriate to simply regress the cell wall digestion‐responsive genes out of previous and future scRNA‐seq stress studies; therefore, we recommend transcriptome fixation during protoplast isolation when aiming to compare subtle transcriptomic changes between treatments or genotypes. In summary, we proved the efficacy of transcriptome fixation to preserve stress response in single cells.

Our analysis also revealed two distinct mesophyll cell populations exhibiting contrasting responses to drought stress, a finding enabled by the transcriptome fixation. The first population, located at the margin of the leaf and around the vasculature, exhibited a typical drought response, consisting of genes induced by ABA, *COLD‐REGULATED* (*COR*) genes and/or *EARLY‐RESPONSIVE TO DEHYDRATION* (*ERD*) genes, such as *ERD12*, *KIN1*, *COR15A*, *COR47, BGLU18* and *LTI78* (Wang *et al*., 1995; Kim & Nam, 2010; Doner *et al*., 2021; Wu *et al*., 2023). We conducted whole‐mount *in situ* hybridizations with probes targeting transcripts of *TSA1* and *BGLU18*, two of the most specific genes marking this population, to localize the cells with this typical drought response within the leaf. *BGLU18* is a β‐glucosidase that hydrolyzes ABA‐glucose ester (inactive form of ABA) to release free ABA in response to stress (Lee *et al*., 2006). *BGLU18* was previously shown to localize in leaf petioles, primarily in endoplasmic reticulum (ER) bodies, which are induced under stress, and to be involved in the early stages of ABA accumulation (Han *et al*., 2020). In fact, *TSA1*, which was reported to facilitate the formation of ER bodies in *Brassicales* (Geem *et al*., 2019), was also enriched in the leaf‐margin mesophyll population. In addition, the observed expression pattern of *BGLU18* is consistent with the reported distribution of ER bodies in Arabidopsis rosette leaves (Nakazaki *et al*., 2019). Furthermore, under drought stress, BGLU18 was found to co‐immunoprecipitate with the phloem‐localized METACASPASE 3 (MC3), a positive regulator of drought response also transcriptionally inducing *BGLU18* (Pitsili *et al*., 2023). In addition, *LTI78* (also named *RESPONSE TO DESSICATION 29A, RD29A*), which is also enriched in this mesophyll population, is induced upon drought, high salinity and low temperature in Arabidopsis cotyledons (Nordin *et al*., 1993; Lee *et al*., 2010). *lti78* mutants were found to be more susceptible to drought, suggesting that LTI78 acts as a positive regulator of the drought stress response, avoiding plant death under severe drought (Liu *et al*., 2020; W. Liu *et al*., 2023). Supporting this, *LTI78* expression is also upregulated by MC3, as well as by ABA, which was proposed to occur through the ERF transcription factor RAP2.6, a target of OPEN STOMATA1 (OST1; Zhu *et al*., 2020; Ndathe *et al*., 2022; Pitsili *et al*., 2023). In this study, transcripts of the genes *TSA1* and *BGLU18*, which both coexpressed with *LTI78*, accumulated at the border of the leaf and in the phloem of the midvein upon drought. Similarly, the promoter of *COR15A*, which is also involved in ABA‐dependent responses to environmental stresses and also targeted by RAP2.6 in the OST1 pathway, is induced upon drought at the border of the leaf and midvein (Gilmour *et al*., 1998, 2004; Zhu *et al*., 2020). In addition, we also observed *COR15A* expression in the mesophyll cells around the vasculature and at the tip of the leaf. Thus, *COR15A* is expressed in more widespread regions of the leaf than *BGLU18* and *TSA1*, but it remains unclear whether this broader expression is reflective of the wider expression partner displayed in our atlas or if it is influenced by the different techniques used for visualization (reporter line vs *in situ* hybridization).

A second transcriptionally distinct mesophyll population responding to drought was localized in the more central, possibly distal, zone of the leaf. This population shared some drought responses with the population described above, but was characterized by an enrichment of genes previously associated with iron starvation or iron homeostasis, such as multiple members of the basic helix–loop–helix (bHLH) transcription factor family (*bHLH38*, *bHLH39*, *bHLH100* and *bHLH101*), *FERRIC REDUCTION OXIDASE3* (*FRO3*) and *FEP1* (Yuan *et al*., 2008; Sivitz *et al*., 2012; Kobayashi *et al*., 2014; Rasheed *et al*., 2016; Kurt & Filiz, 2018; Muhammad *et al*., 2022). In addition, by inferring gene regulatory networks across the different mesophyll populations, some of these genes (*bHLH38*, *bHLH39* and *bHLH100*) were identified as candidate transcription factors specifically enriched in the cells within the iron starvation response mesophyll population, further suggesting their importance in the regulation of the response. However, it remains to be studied whether the observed changes in the expression of iron starvation‐related genes could be attributed to a reduction in iron availability upon drought, or whether the genes are part of a drought response not, or not directly, related to the iron levels in these cells. Iron is an essential micronutrient for various cellular processes, including photosynthesis and respiration. Drought has previously been shown to impact iron homeostasis in sorghum roots, possibly to protect root cells against reactive oxygen species (Kim *et al*., 2019; L. Xu *et al*., 2021). In addition, iron application has been shown to enhance drought tolerance in maize, canola and wheat (Tripathi *et al*., 2018; Rezayian *et al*., 2023; Abdullah *et al*., 2024; Mazhar *et al*., 2024). However, the precise molecular and functional connections between drought and iron homeostasis are still not well understood. Future studies are required to elucidate the potential link between drought and iron starvation responses in the leaf mesophyll at the molecular level.

In conclusion, we identified two mesophyll populations displaying different transcriptional responses to MD stress. First, we identified a drought‐responsive population of mesophyll cells relatively distant from the vasculature expressed genes previously associated with iron starvation. Second, we identified mesophyll cells at the borders and midvein that showed early ABA responses, possibly serving as the first line of defense against drought or the initial recipients of water starvation signals from the root. The identification of two distinct mesophyll cell populations with different stress responses highlights the complexity of plant adaptation to drought, providing new insights into the tissue‐ or in this case within‐tissue‐specific responses underlying plant responses to MD.

### Limitations of the study

In this study, among other questions, we addressed whether cell wall digestion and the simultaneous treatment with a transcription inhibitor trigger transcriptome changes in different cell types. Several conclusions about this were drawn by comparing the data of samples that underwent cell wall digestion, either in or not in the presence of the transcription inhibitor, with that of undigested leaves. However, such interpretations are not straightforward because other factors – besides cell type sensitivity to protoplast generation or to the transcription inhibitor – can cause bias. For example, we have limited knowledge about whether certain types of cells are digested more easily than others and, thus, ultimately become over‐ or under‐represented in the Digested samples. To correct for this, an appropriate control would be a single‐cell transcriptomic profile of leaves that has been obtained from intact cells (not nuclei) without cell wall digestion – which is currently technically not feasible. Several conclusions from this study are, thus, limited by the absence of such a control. First, the comparisons between the bulk RNA‐seq and scRNA‐seq data are limited by differences in cell composition of the Digested samples caused by the isolation process, which might not be reflecting the true cellular composition of the leaf. Consequently, as the cell wall digestion score was derived from bulk RNA‐seq data of Digested samples and subsequently applied to single‐cell data, it might introduce a bias toward the cell types that were most easily digestible. Second, comparisons between transcript levels of the Undigested vs Fixed and Digested samples are limited in a similar manner, as we cannot rule out that ActD affects different cell populations unevenly. Finally, as we compared samples treated with a transcription inhibitor (Fixed–Digested samples) with samples without, the transcript levels might be affected by mRNA degradation rates, causing potential bias. Despite these limitations, this study demonstrates that applying a transcription inhibitor during protoplast generation improves the study of MD responses by limiting the impact of cell wall digestion on the transcriptome.

## Competing interests

None declared.

## Author contributions

RTB and MD conceptualized the work. RTB, EV, TE, CG, LDV, BDR and MD conceived the experiments. RTB, EV, TE and MD performed the experiments. RTB, TE and MD performed data analysis. TE, RTB and MD conceived the data visualization tool. BDR, LDV and MD supervised the project. RTB and MD drafted the manuscript which was further improved by all authors.

## Disclaimer

The New Phytologist Foundation remains neutral with regard to jurisdictional claims in maps and in any institutional affiliations.

## Supporting information


**Fig. S1** Gene expression changes upon cell wall digestion of Arabidopsis leaves.
**Fig. S2** Functional analysis of gene expression changes caused by cell wall digestion of Arabidopsis leaves.
**Fig. S3** Comparison of the transcriptome profiles of Digested and Fixed–Digested samples with the Undigested sample.
**Fig. S4** Gene expression changes in Arabidopsis leaves upon mild drought.
**Fig. S5** Differential gene expression analysis upon mild‐drought treatment of Arabidopsis.
**Fig. S6** Expression profiles of genes with significant interaction between the growth condition and the cell isolation method.
**Fig. S7** Quality control of the Arabidopsis leaf scRNA‐seq samples.
**Fig. S8** Droplet rate scores of the Arabidopsis leaf scRNA‐seq samples.
**Fig. S9** Transcriptional variation in the Arabidopsis leaf single‐cell datasets.
**Fig. S10** Cell wall digestion response score in the Arabidopsis leaf scRNA‐seq dataset.
**Fig. S11** Quality control of the curated Arabidopsis leaf scRNA‐seq dataset.
**Fig. S12** Main tissue populations and cell states in the curated Arabidopsis leaf scRNA‐seq dataset.
**Fig. S13** Main tissue populations, cell states and annotation of remaining Arabidopsis leaf scRNA‐seq datasets.
**Fig. S14** Tissue‐specific and shared responses to cell wall digestion of Arabidopsis leaves.
**Fig. S15** Expression of additional tissue‐specific marker genes.
**Fig. S16** GUS staining images for the reporter lines of each Arabidopsis leaf tissue.
**Fig. S17** Additional confocal microscopy images for the reporter lines of each Arabidopsis leaf tissue.
**Fig. S18** Tool for single‐cell data visualization in an Arabidopsis leaf section.
**Fig. S19** Mild‐drought responses shared between Arabidopsis leaf tissues.
**Fig. S20** Expression of drought‐related genes in the Arabidopsis mesophyll tissue.
**Fig. S21** Expression of *BGLU18* in the combined Arabidopsis leaf scRNA‐seq dataset.
**Fig. S22** Transcript visualization of canonical drought responses in Arabidopsis leaves.
**Fig. S23** Expression of *BGLU18* and *TSA1* in the Arabidopsis leaf single‐cell dataset of the Fixed–Digested samples.
**Fig. S24** Coexpression of dual drought responses in the Arabidopsis mesophyll.
**Fig. S25** Microscopic images used for image analysis of the dual drought response.
**Fig. S26** Quantification of the dual fluorescent signal.
**Fig. S27** Regulatory map (regmap) output of the MINI‐EX analysis.
**Fig. S28** Expression of tissue‐specific marker genes in the combined Arabidopsis leaf scRNA‐seq dataset.

**Table S1** Primer list.

**Table S2** Cell wall digestion response in Arabidopsis leaves and gene clustering.

**Table S3** Information and metadata parameters of the scRNA‐seq dataset.

**Table S4** Bulk RNA‐seq of Arabidopsis leaf samples: normalized data.

**Table S5** Drought responses per sample type used in the Arabidopsis leaf scRNA‐seq experiments.

**Table S6** Interactions between drought and cell wall digestion responses in Arabidopsis leaves.

**Table S7** Pseudobulk analysis of cell wall digestion responses in Arabidopsis leaves.

**Table S8** Drought responses per Arabidopsis leaf tissue and functional analysis.

**Table S9** Drought‐responsive genes in the Arabidopsis leaf mesophyll.
Please note: Wiley is not responsible for the content or functionality of any Supporting Information supplied by the authors. Any queries (other than missing material) should be directed to the *New Phytologist* Central Office.

## Data Availability

Raw and processed data of the scRNA‐seq experiments can be accessed at NCBI with respective GEO no.: GSE273033, together with the technical information following the guidelines of Grones *et al*. (2024; Table S3). An online tool to visualize the scRNA‐seq data is available at https://single‐cell.be/plant/leaf‐drought. The raw bulk RNA‐seq data can be accessed at NCBI with GEO no.: GSE273926.
