# Supplementary material for: Dual and spatially resolved drought responses in the Arabidopsis leaf mesophyll revealed by single‐cell transcriptomics
Source: New Phytol. 2025 Mar 3;246(3):840–58. doi: 10.1111/nph.20446 (PMC11982798; doi:10.1111/nph.20446)
Supplement: Supplementary file 1 — Fig. S1 Gene expression changes upon cell wall digestion of Arabidopsis leaves. Fig. S2 Functional analysis of gene expression changes caused by cell wall digestion of Arabidopsis leaves. Fig. S3 Comparison of the transcriptome profiles of Digested and Fixed–Digested samples with the Undigested sample. Fig. S4 Gene expression changes in Arabidopsis leaves upon mild drought. Fig. S5 Differential gene expression analysis upon mild‐drought treatment of Arabidopsis. Fig. S6 Expression profiles of genes with significant interaction between the growth condition and the cell isolation method. Fig. S7 Quality control of the Arabidopsis leaf scRNA‐seq samples. Fig. S8 Droplet rate scores of the Arabidopsis leaf scRNA‐seq samples. Fig. S9 Transcriptional variation in the Arabidopsis leaf single‐cell datasets. Fig. S10 Cell wall digestion response score in the Arabidopsis leaf scRNA‐seq dataset. Fig. S11 Quality control of the curated Arabidopsis leaf scRNA‐seq dataset. Fig. S12 Main tissue populations and cell states in the curated Arabidopsis leaf scRNA‐seq dataset. Fig. S13 Main tissue populations, cell states and annotation of remaining Arabidopsis leaf scRNA‐seq datasets. Fig. S14 Tissue‐specific and shared responses to cell wall digestion of Arabidopsis leaves. Fig. S15 Expression of additional tissue‐specific marker genes. Fig. S16 GUS staining images for the reporter lines of each Arabidopsis leaf tissue. Fig. S17 Additional confocal microscopy images for the reporter lines of each Arabidopsis leaf tissue. Fig. S18 Tool for single‐cell data visualization in an Arabidopsis leaf section. Fig. S19 Mild‐drought responses shared between Arabidopsis leaf tissues. Fig. S20 Expression of drought‐related genes in the Arabidopsis mesophyll tissue. Fig. S21 Expression of BGLU18 in the combined Arabidopsis leaf scRNA‐seq dataset. Fig. S22 Transcript visualization of canonical drought responses in Arabidopsis leaves. Fig. S23 Expression of BGLU18 and TSA1 in the Arabidopsis leaf si [file NPH-246-840-s005.pdf]

New Phytologist Supporting Information

Article title: Dual and spatially resolved drought responses in the Arabidopsis leaf mesophyll revealed by single-cell transcriptomics
Authors: Rubén Tenorio Berrío, Eline Verhelst, Thomas Eekhout, Carolin Grones, Lieven De Veylder, Bert De Rybel, Marieke Dubois
Article acceptance date: 13 January 2025

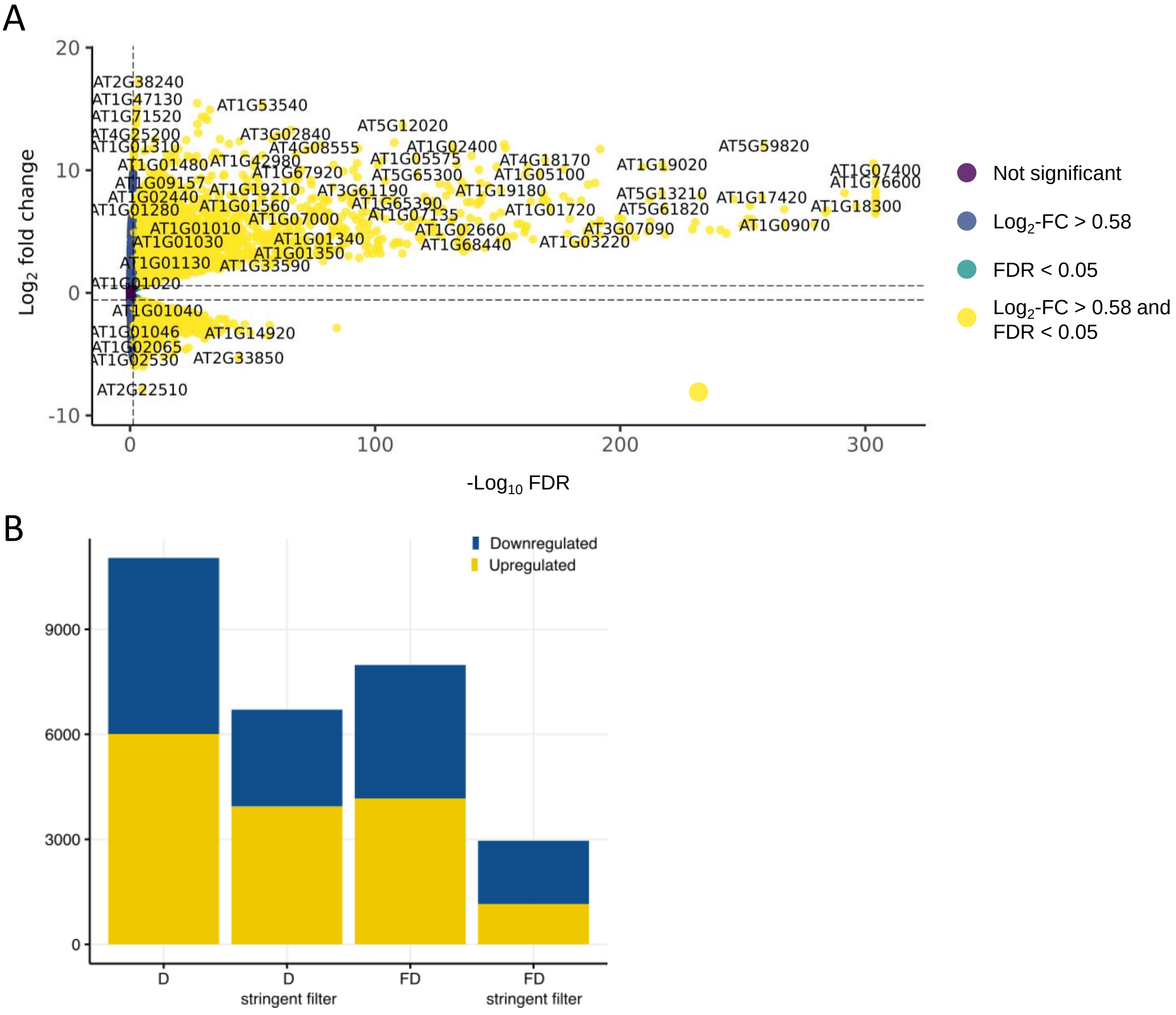

Supp. Figure 1. Gene expression changes upon cell wall digestion of Arabidopsis leaves. (A) Volcano plot of the cell wall digestion triggered response, using only False Discovery Rate (FDR) < 0.05 as a threshold for significance. **(B)** Stacked bar plot displaying the number of upregulated (yellow) and downregulated (blue) genes upon cell wall digestion in the digested (D) and Fixed-Digested (FD) samples compared to the undigested (U) leaf. Less (FDR < 0.05) or more (Log_2 FC > 1; FDR < 0.01) stringent filters were applied.

A

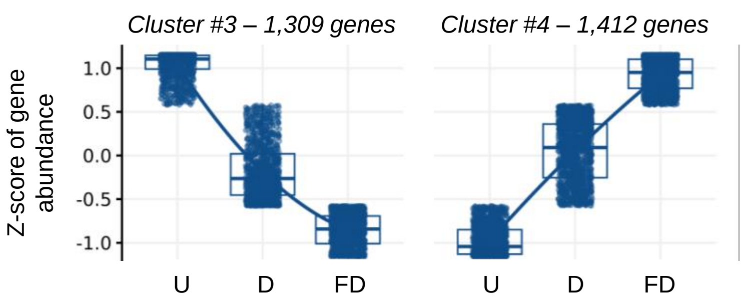

B

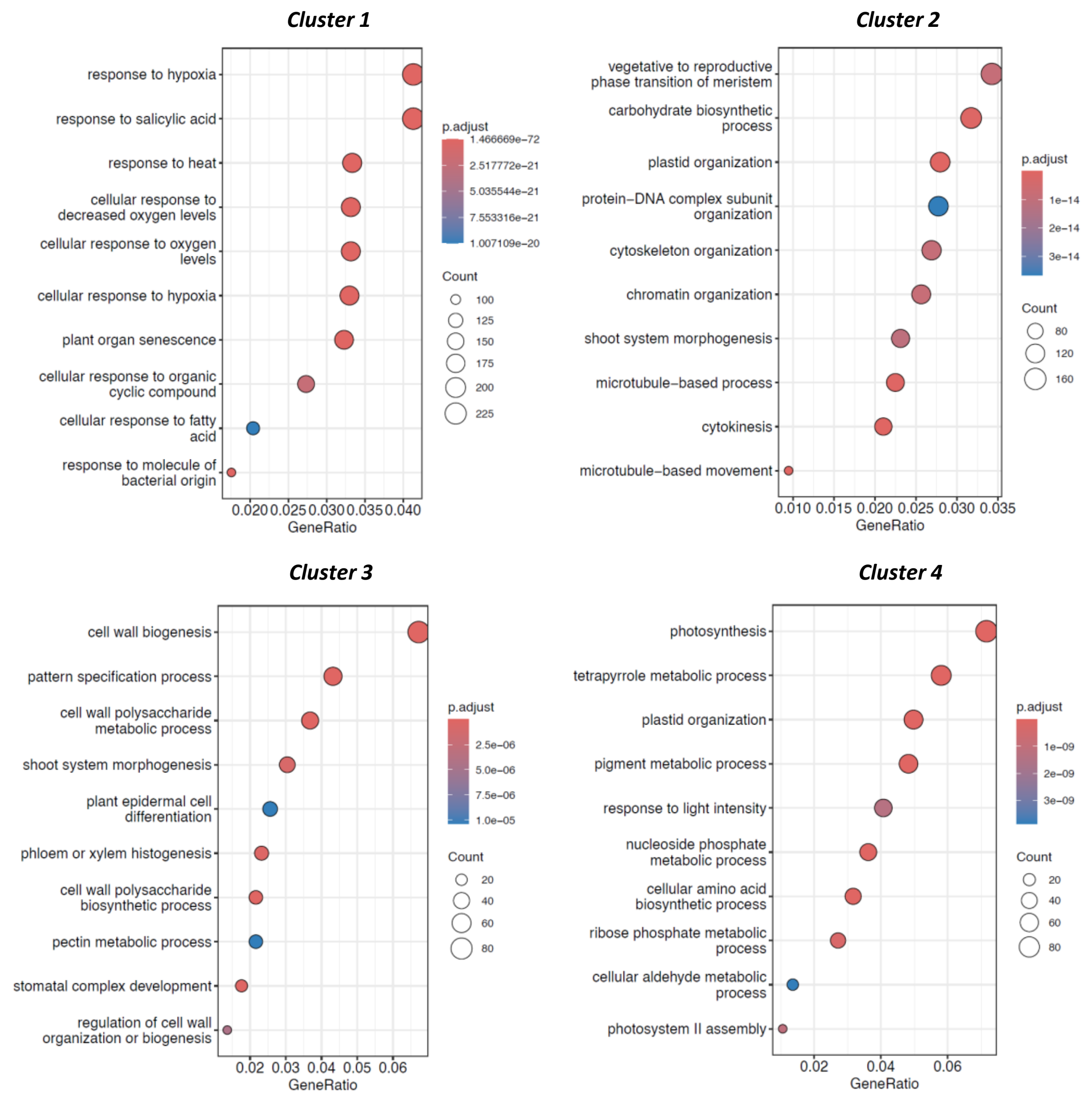

Supp. Figure 2. Functional analysis of gene expression changes caused by cell-wall digestion of Arabidopsis leaves. (A) k-means clustering performed on the differentially expressed genes captured upon cell-wall digestion without (D) and with (FD) fixation, compared to the undigested leaf tissue (U). The less abundant gene clusters are shown, while the most abundant clusters are shown in Figure 1C. The boxplot delineates the 25%-75% interval and the horizontal line represents the median. **(B)** Dotplots displaying the most enriched Biological Process GO terms in the different clusters of genes affected by cell-wall digestion (Cluster 1 and 2 shown in Figure 1C, Cluster 3 and 4 shown here in (A)).

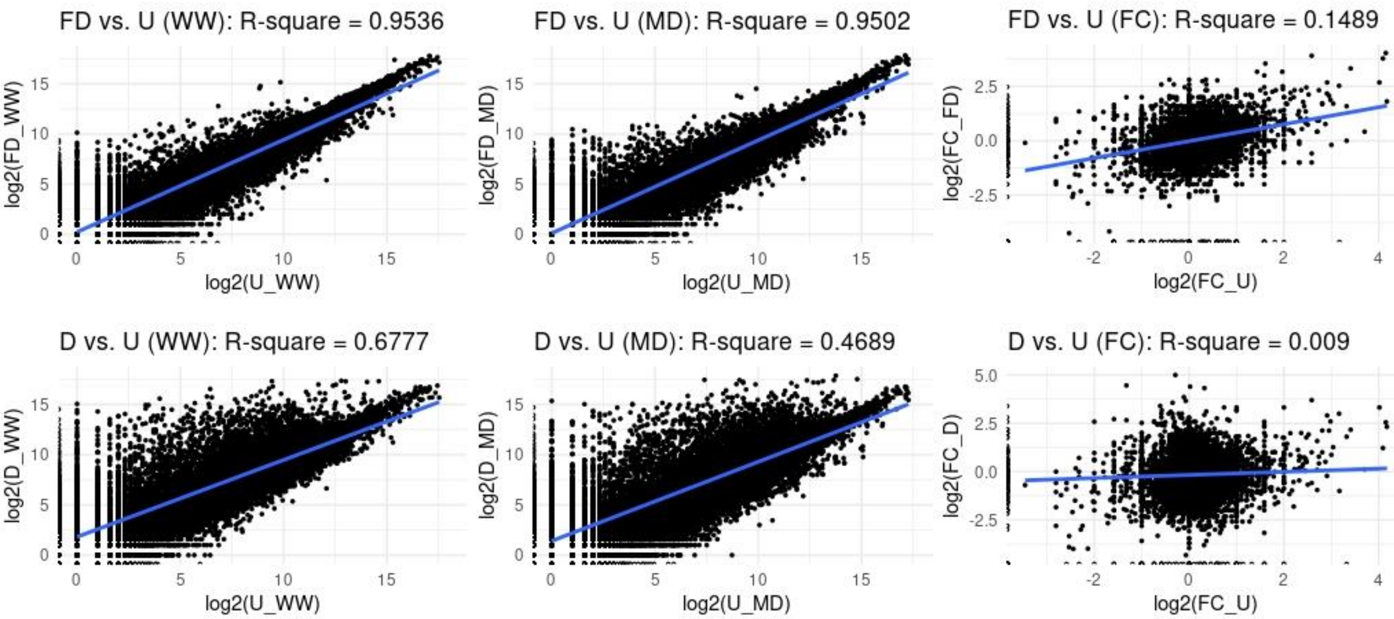

Supp. Figure 3. Comparison of the transcriptome profiles of Digested and Fixed-Digested samples with the Undigested sample. Correlation plots comparing the normalized level of each transcript between the Digested (D) or Fixed-Digested (FD) samples against the Undigested (U) leaf in the well-watered (WW) and mild drought-treated (MD) Arabidopsis samples. The right panels display the correlation in the Log_2 fold change (FC) between the MD and WW samples.

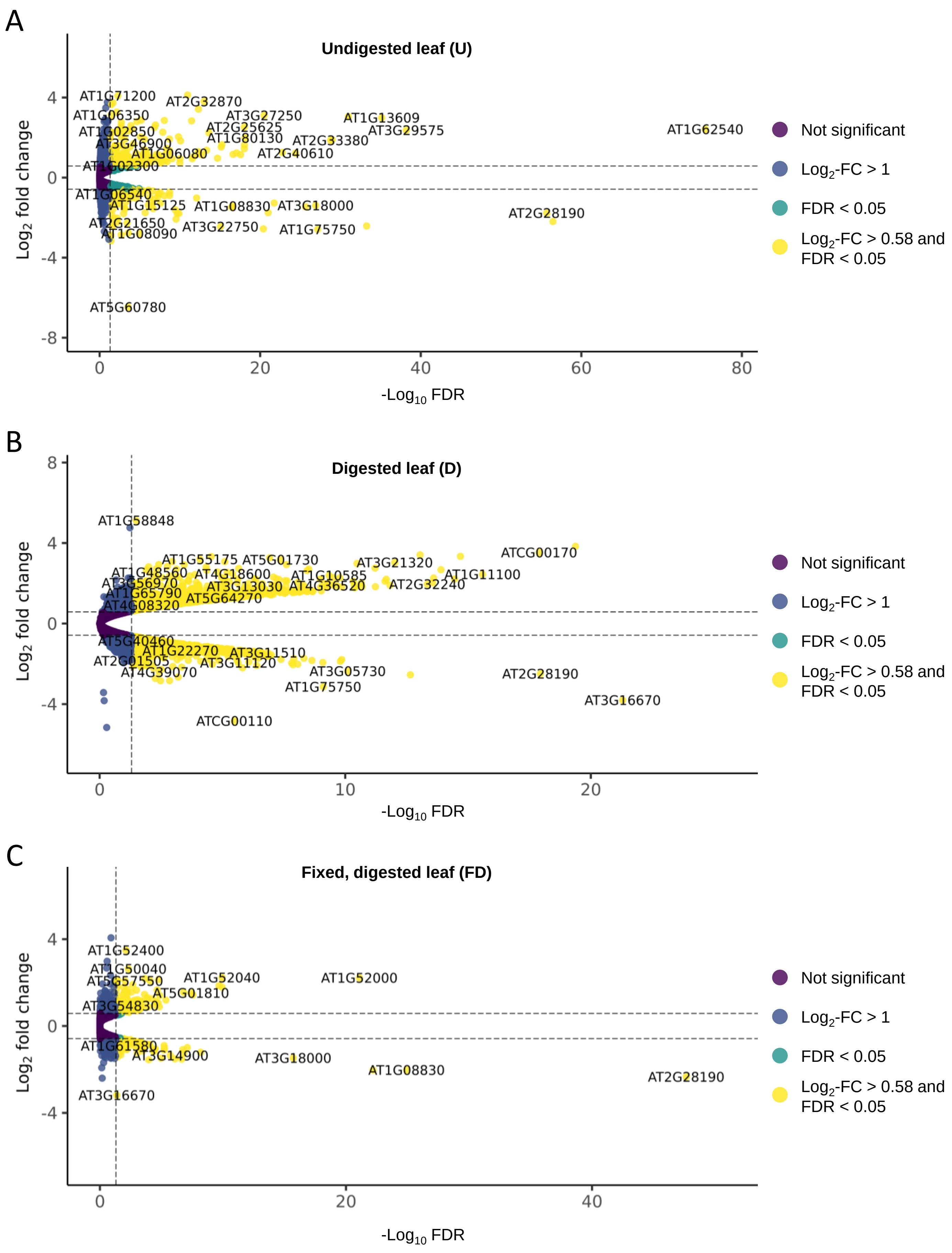

Supp. Figure 4. Gene expression changes in Arabidopsis leaves upon mild drought. Volcano plots of drought-triggered response in undigested leaves **(A)**, digested leaves **(B)** and fixed and digested leaves **(C)**, using False Discovery Rate (FDR) < 0.05 and Log_2 Fold Change (FC) > 1 as thresholds for significance.

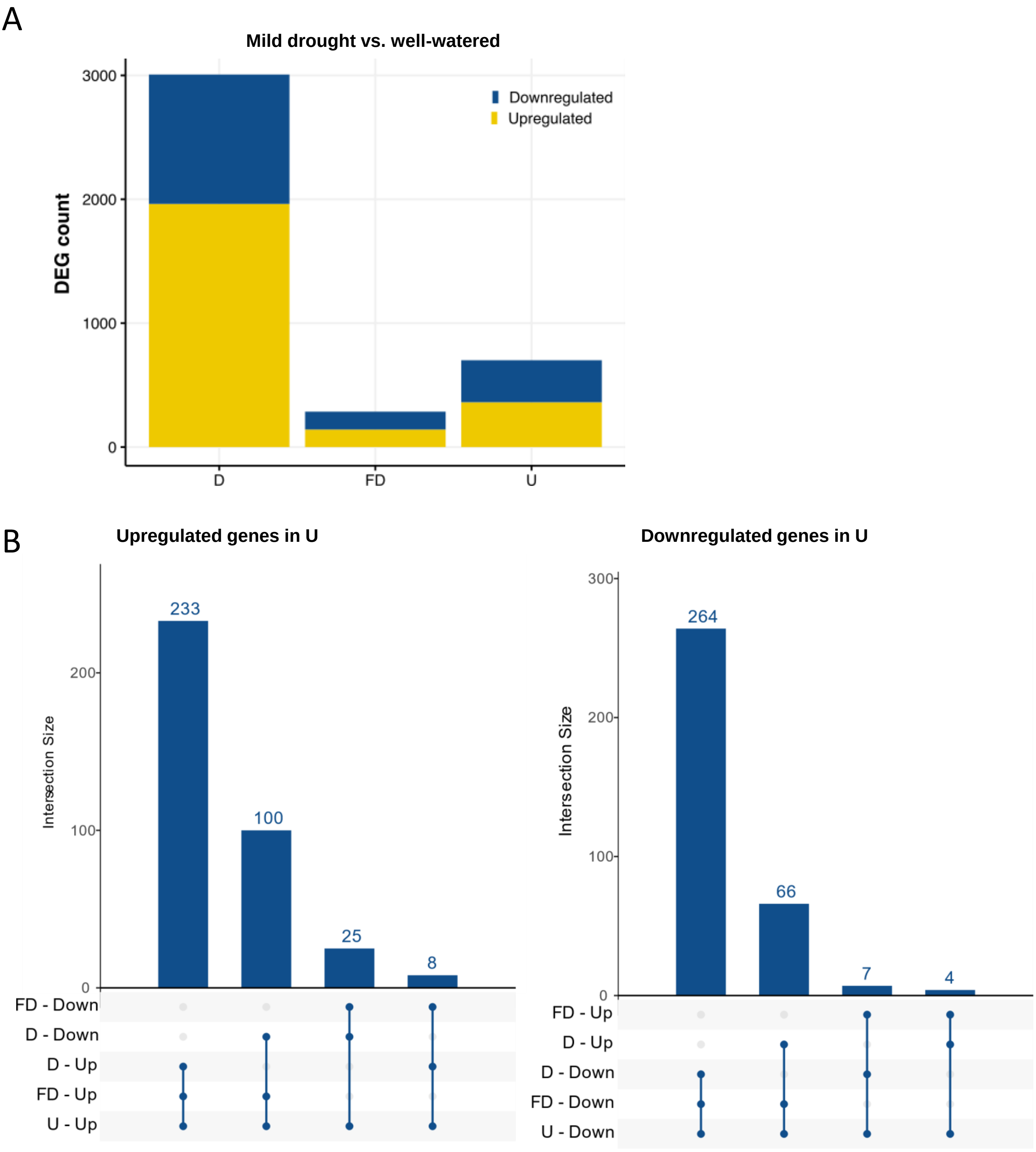

Supp. Figure 5. Differential gene expression analysis upon mild drought treatment of Arabidopsis. (A) Stacked bar plot displaying the number of upregulated (yellow) and downregulated (blue) differentially expressed genes (DEG) upon mild-drought treatment in the Undigested (U), Digested (D) and Fixed-Digested (FD) samples (False Discovery Rate < 0.05). **(B)** Upset plot visualization of the behavior of genes upregulated (left) and downregulated (right) upon mild drought in the undigested leaf in the different samples. Bar plots in the upper panel depict the sizes of the intersections indicated in the lower panel.

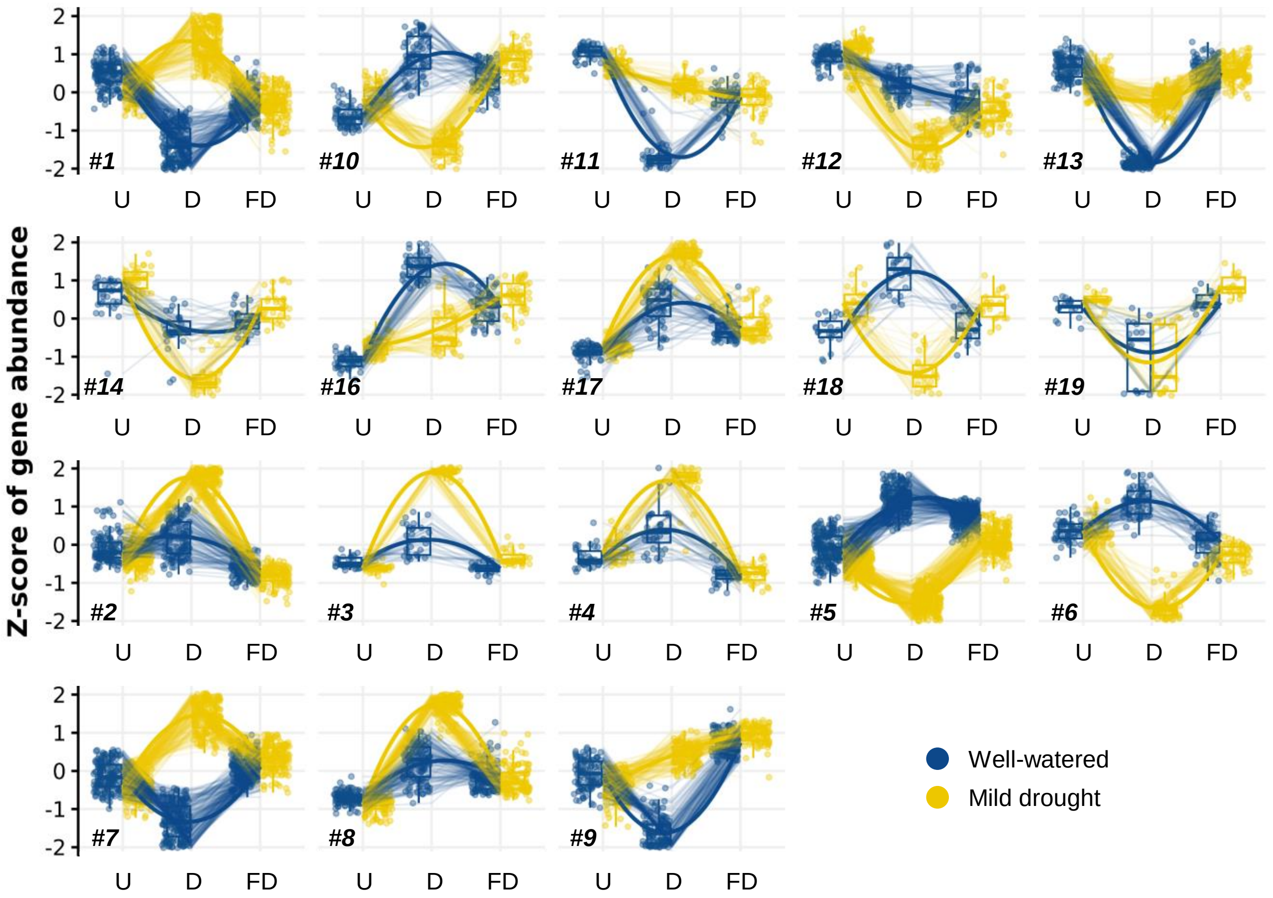

Supp. Figure 6. Expression profiles of genes with significant interaction between the growth condition and the cell isolation method. k-means clustering performed on the differentially expressed genes captured upon interaction analysis between the isolation method (digestion (D), fixation and digestion (FD), or undigested leaves (U)) and the growth condition (well-watered or drought treatment of Arabidopsis). The number in the lower left corner indicates the cluster number. Note that clusters #5 and #7 are also shown in Figure 1F. The list of genes within each cluster can be found in Supp. Table 2. For each sample, a boxplot represents the dispersion (25%-75% interval) of the z-score of gene abundance in each plant growth condition, well-watered or mild drought. The horizontal line within the boxplots represents the median.

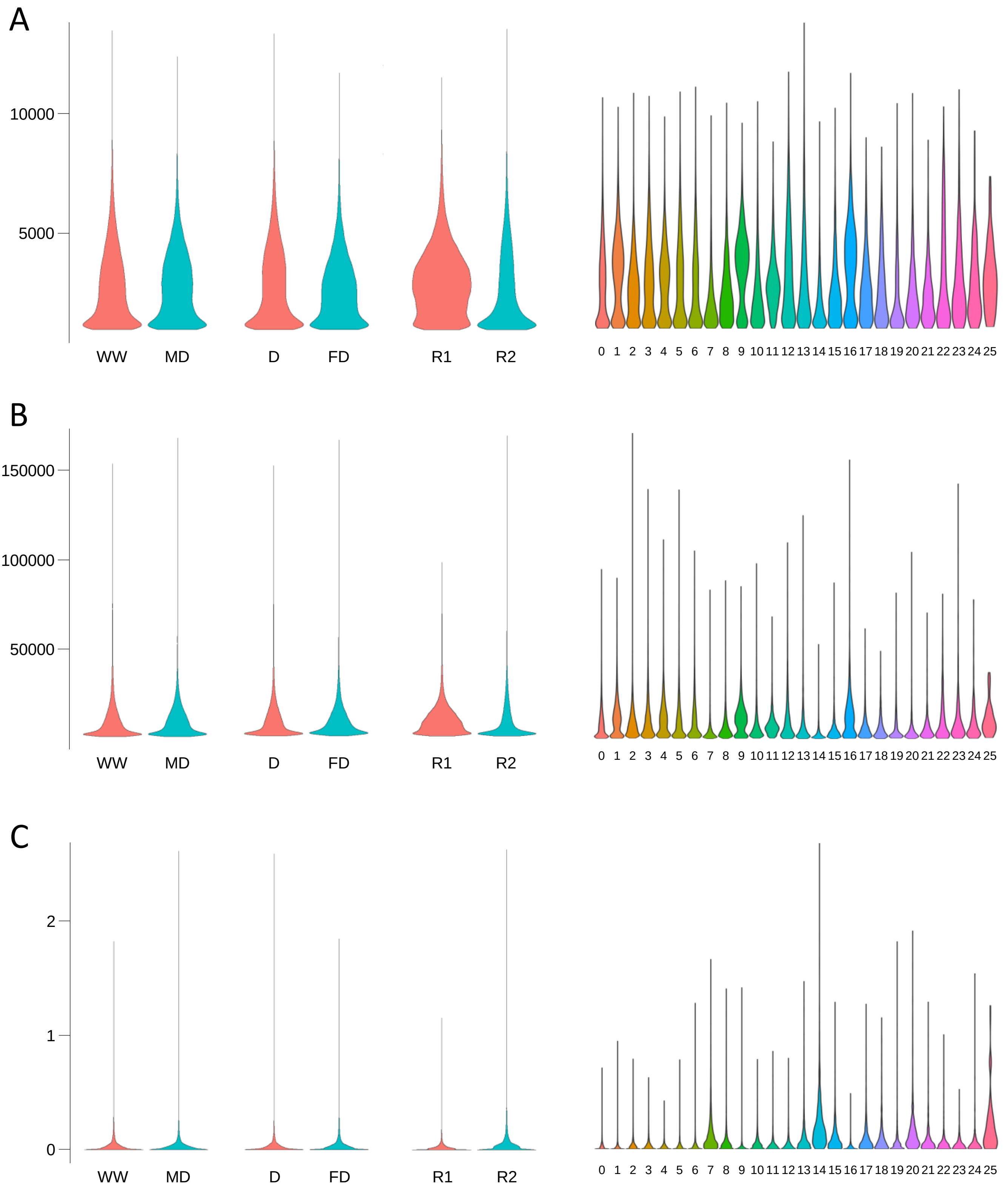

Supp. Figure 7. Quality control of the Arabidopsis leaf scRNA-seq samples. Violin plots representing the number of RNA features **(A)**, the number of counts **(B)** and the percentage of mitochondrial transcripts **(C)**. Data was grouped per condition (WW = well-watered, MD = mild drought), per cell isolation method (D = digested, FD = fixed and digested), per replicate (R = replicate) or per cluster (0-25).

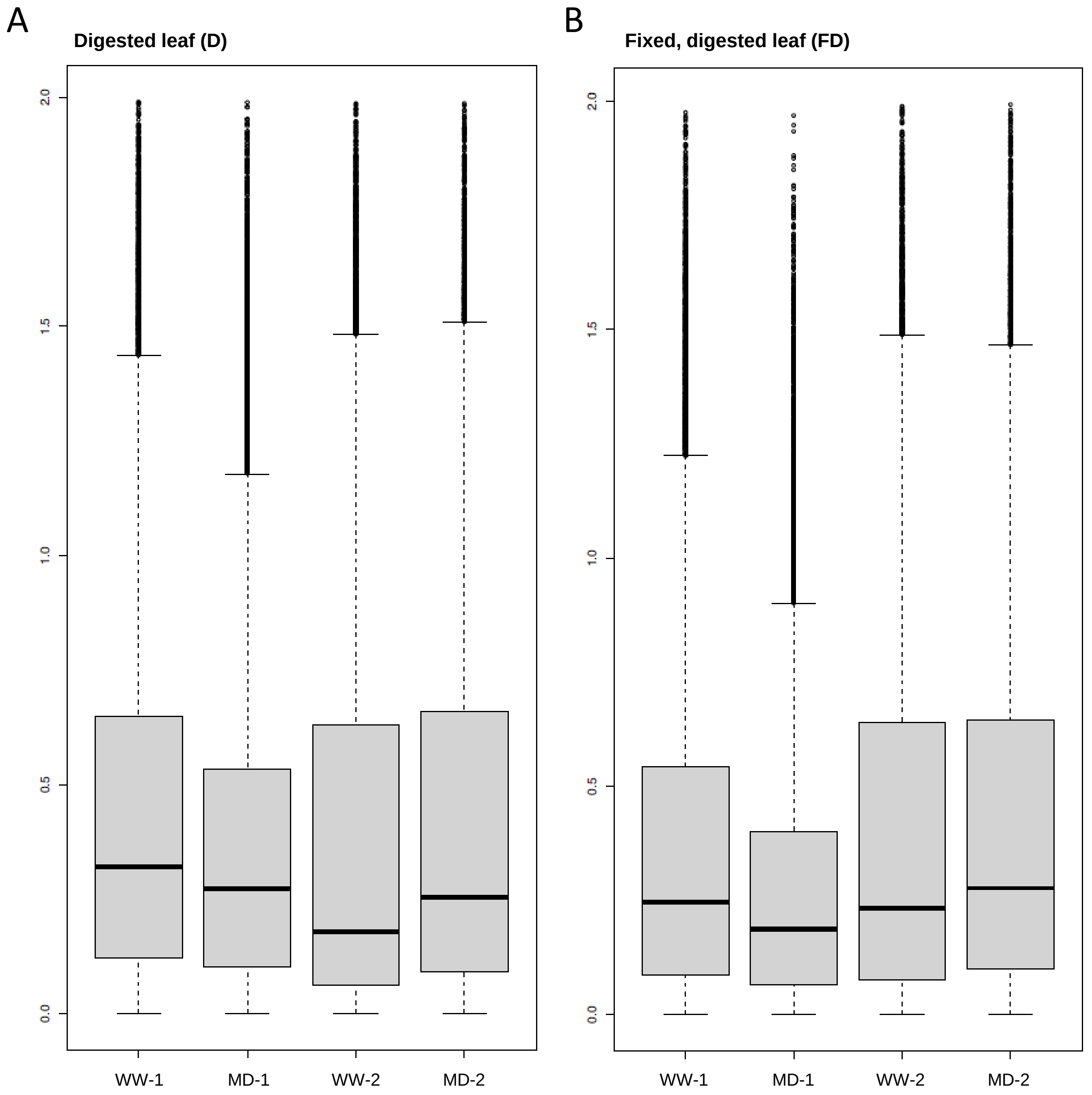

Supp. Figure 8. Droplet rate scores of the Arabidopsis leaf scRNA-seq samples. Boxplot depicting the hybrid droplet score, being a combination of the bcds and cxds methods, grouped by sample in the digested **(A)** and the fixed and digested datasets **(B)**. Boxplots delineate the 25%-75% interval, the whiskers delineate the measurements within 1.5 times the interquartile range and the dots represent outliers. WW = well-watered, MD = mild drought, cxds = co-expression based doublet scoring, bcds = binary classification based doublet scoring.

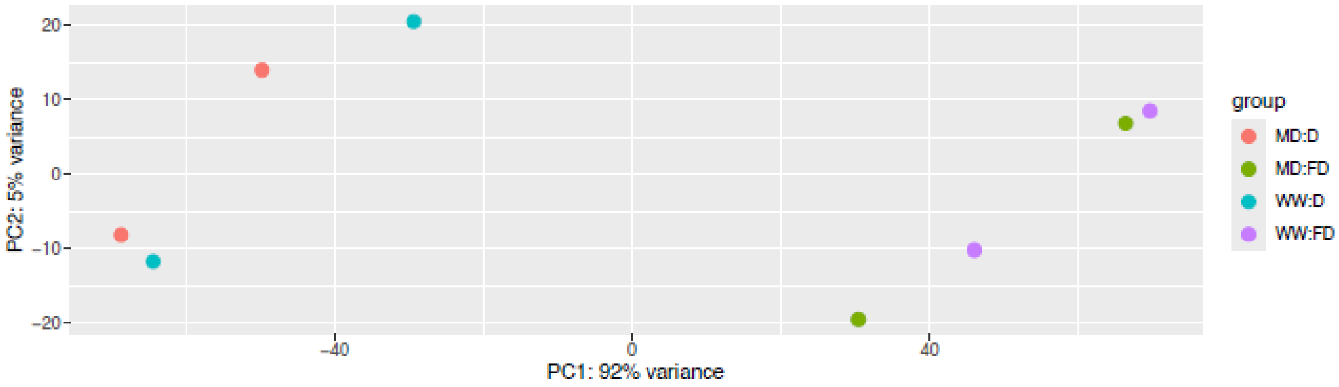

Supp. Figure 9. Transcriptional variation in the Arabidopsis leaf single-cell datasets. Principal component analysis plot depicting the two main principal components (PC1, PC2) of the scRNA-seq samples. MD = mild drought, WW = well-watered, D = Digested, FD = Fixed digested

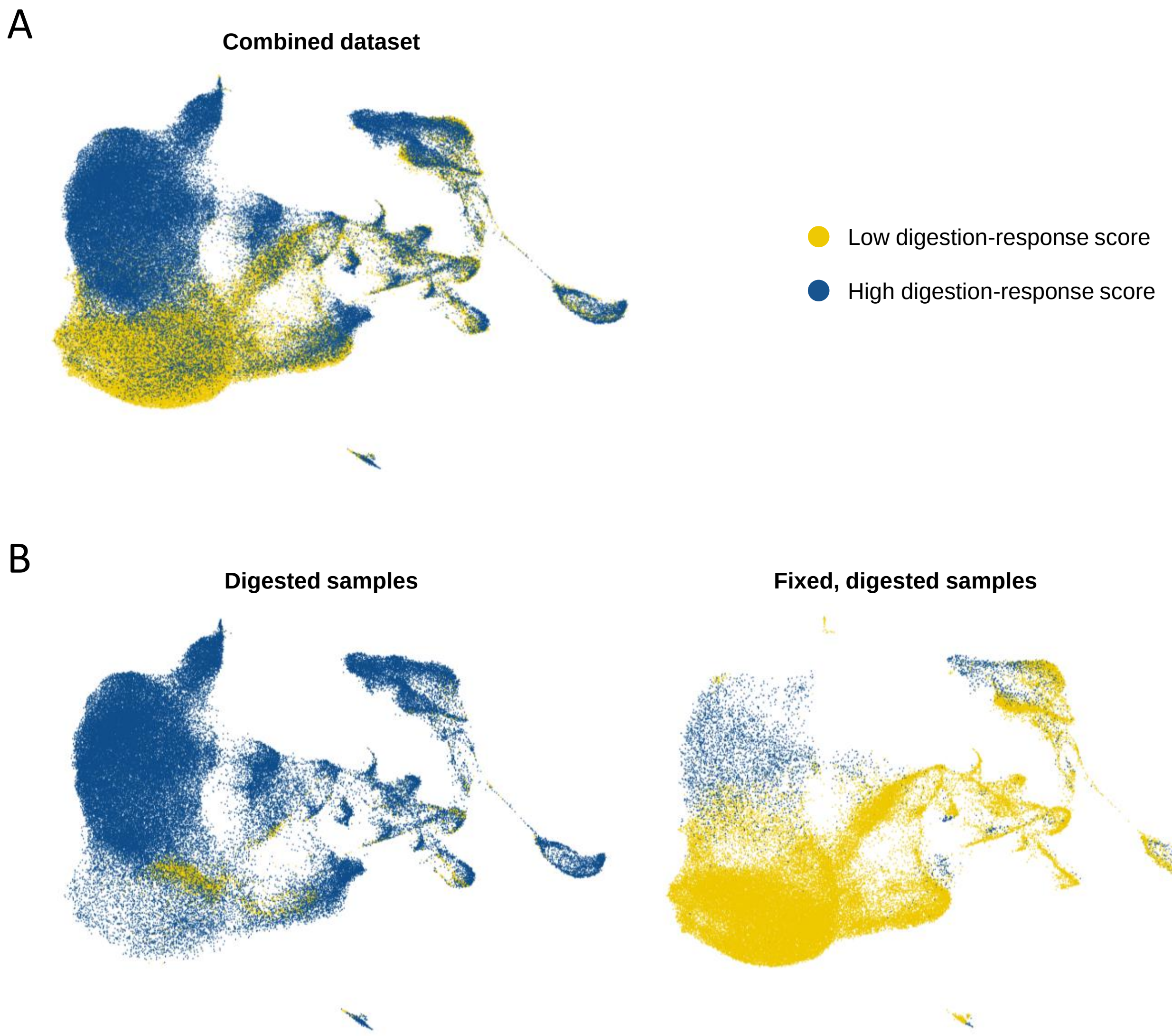

Supp. Figure 10. Cell wall digestion-response score in the Arabidopsis leaf scRNA-seq dataset. UMAP visualization of the combined dataset **(A)** and the dataset split by cell isolation method **(B)** with indication of cells showing a low or high cell wall digestion-response score. This score was calculated based on the top-250 cell wall digestion-responsive genes (calculated from the digested samples, and excluding the genes interacting with the study of mild drought). UMAP = Uniform Manifold Approximation and Projection

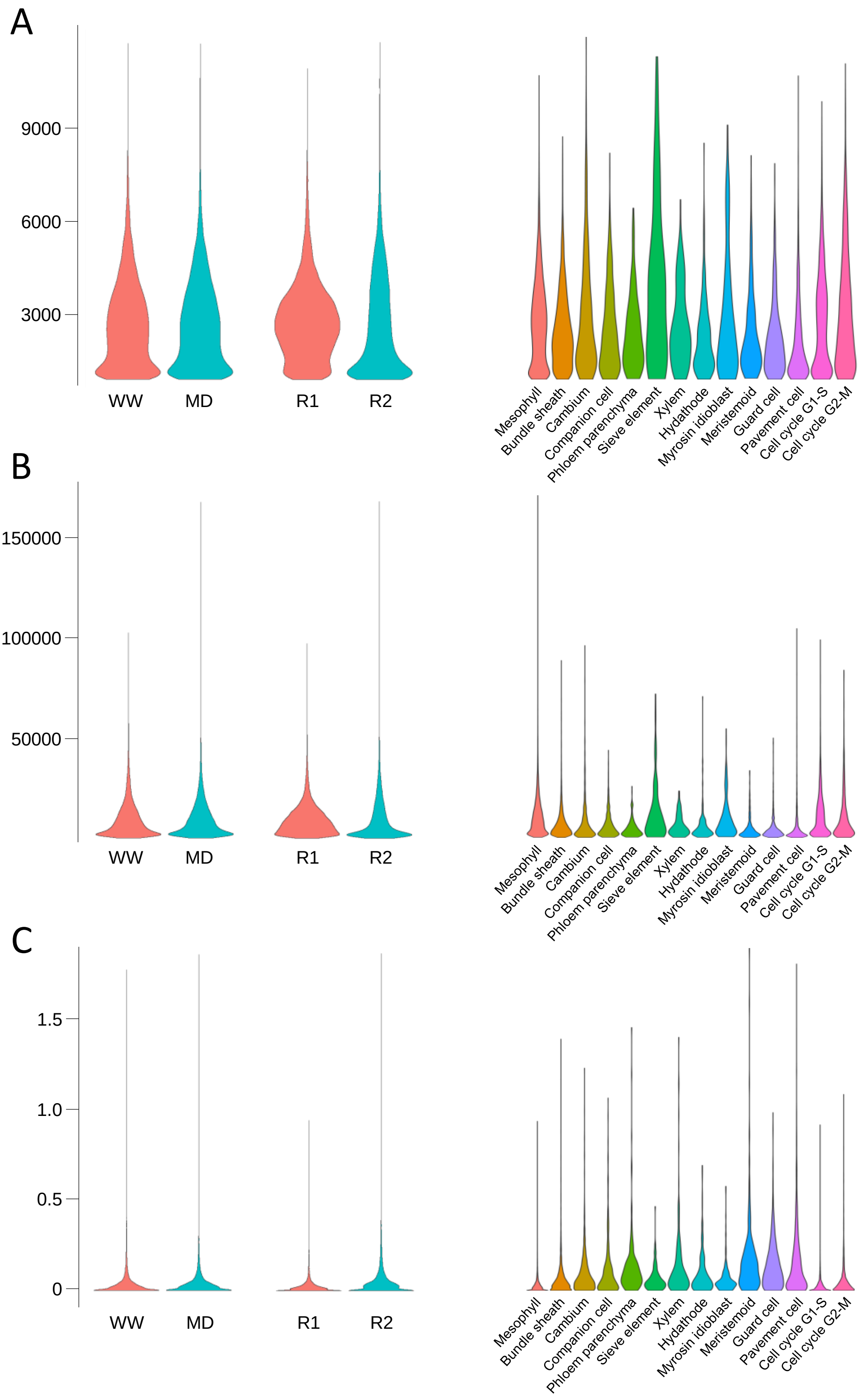

Supp. Figure 11. Quality control of the curated Arabidopsis leaf scRNA-seq dataset. Violin plots representing the number of RNA features **(A)**, the number of counts **(B)** and the percentage of mitochondrial transcripts **(C)**. Data was grouped per condition (WW = well-watered, MD = mild drought), per replicate (R = replicate) or per cell type.

A

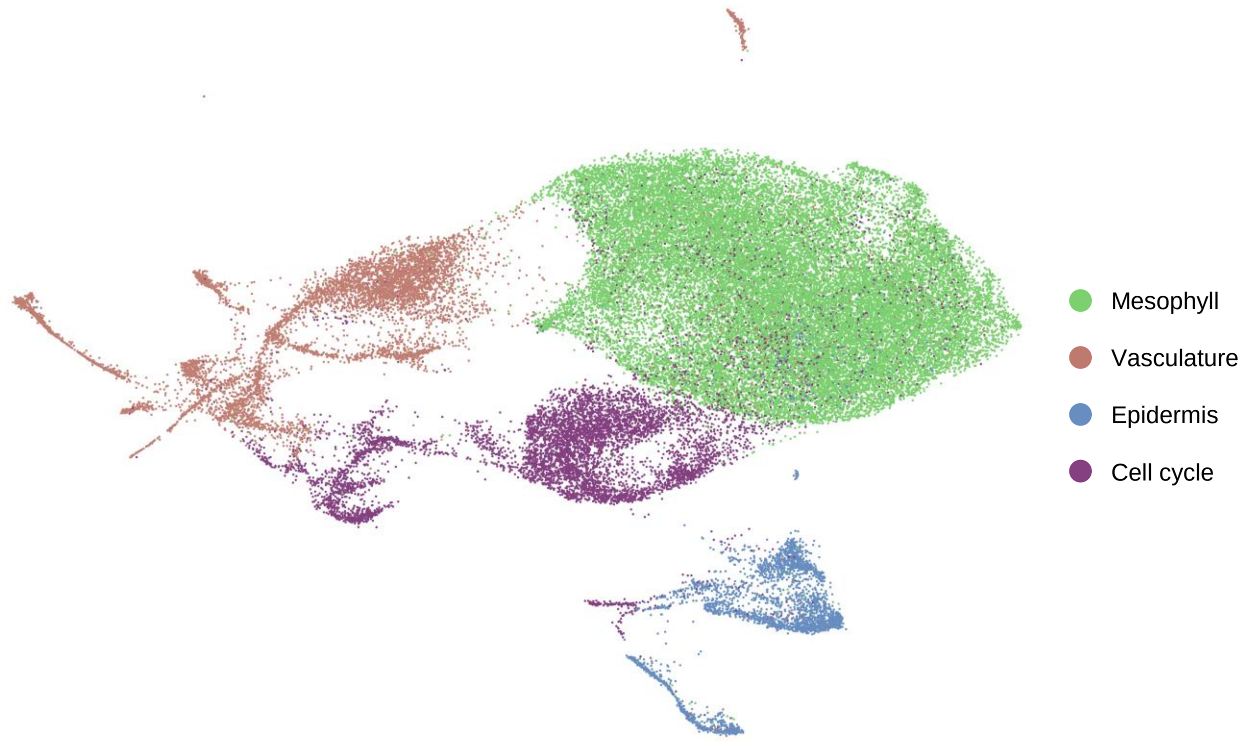

B

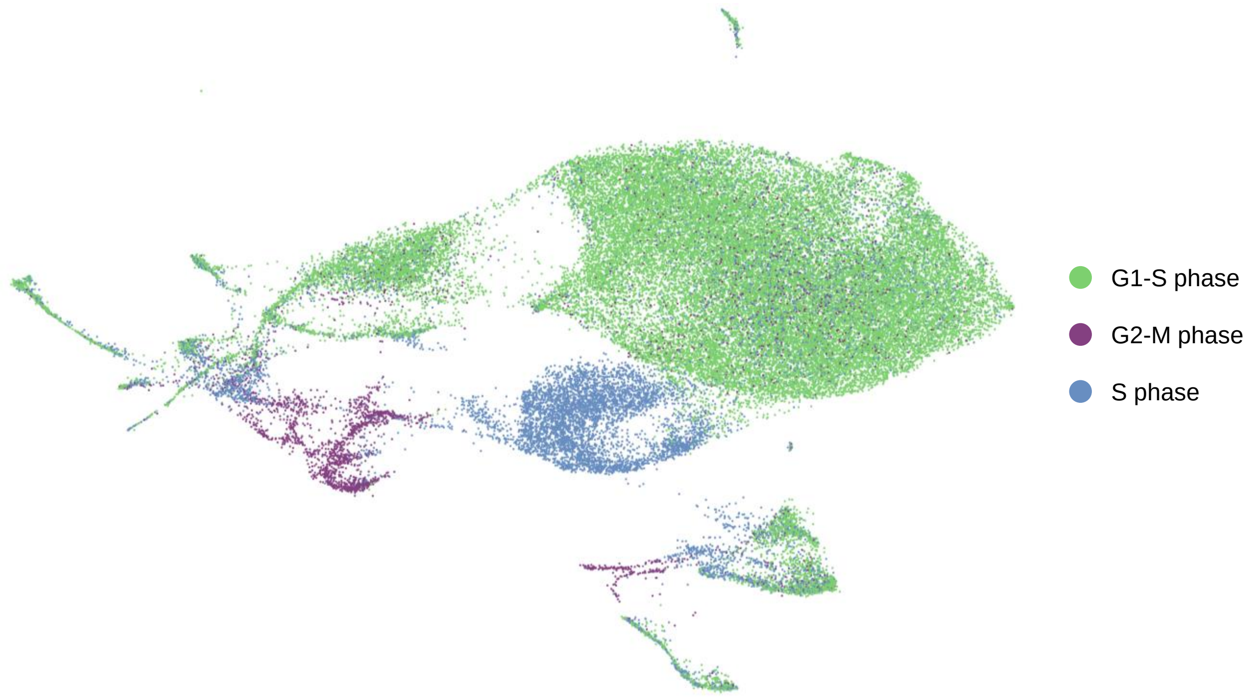

Supp. Figure 12. Main tissue populations and cell states in the curated Arabidopsis leaf scRNA-seq dataset. UMAP plots depicting the curated dataset of fixed and digested samples, colored according to main tissue **(A)** or to cell cycle phase **(B)**. G = Gap, M = Mitosis, S = (DNA) Synthesis, UMAP = Uniform Manifold Approximation and Projection

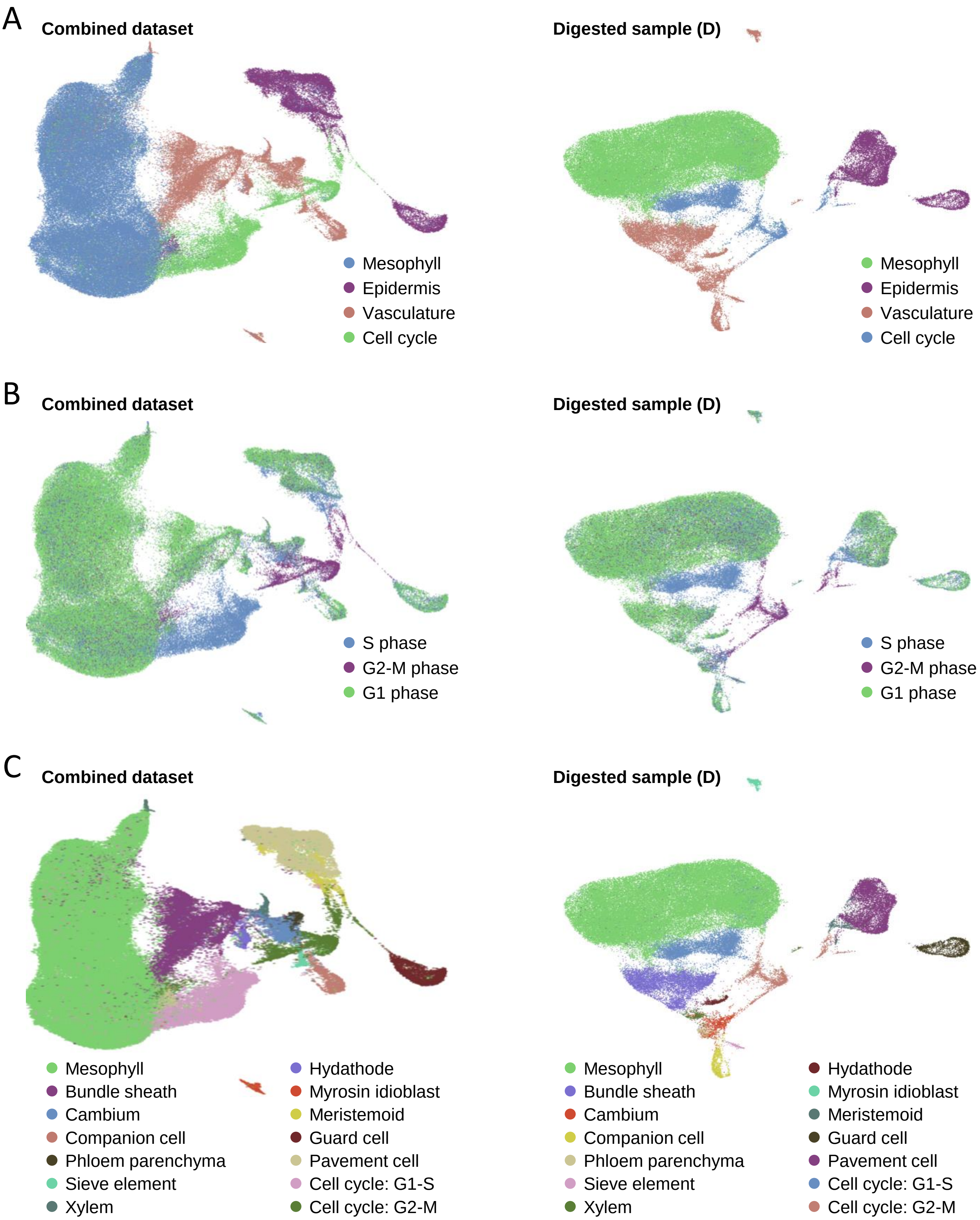

Supp. Figure 13. Main tissue populations, cell states and annotation of remaining Arabidopsis leaf scRNA-seq datasets. UMAP plots depicting the combined dataset (left panels) and the one of digested samples without fixation (right panels), colored according to main tissue **(A)**, to cell cycle phase **(B)** or to cell type/state **(C)**. G = Gap, M = Mitosis, S = (DNA) Synthesis, UMAP = Uniform Manifold Approximation and Projection

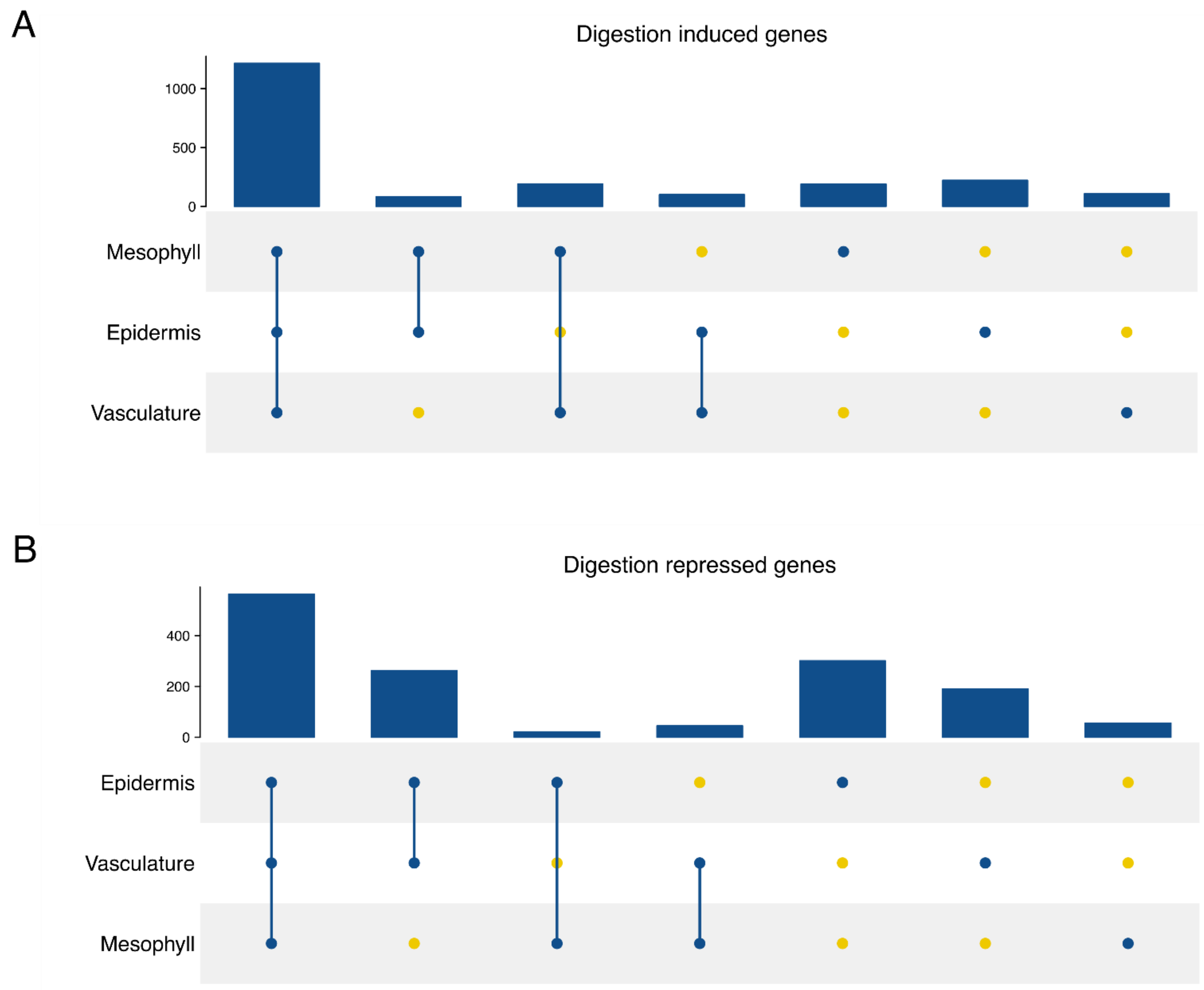

Supp. Figure 14. Tissue-specific and shared responses to cell wall digestion of Arabidopsis leaves. Upset plot visualization of the total number of common genes among the selected main-tissues, depicted in the lower panel, between the genes induced **(A)** or repressed **(B)** upon cell wall digestion. Bar plots in the upper panel depict the sizes of the intersections indicated in blue in the lower panel. Yellow dots indicate the tissues not taken into account for the gene count in the upper panel.

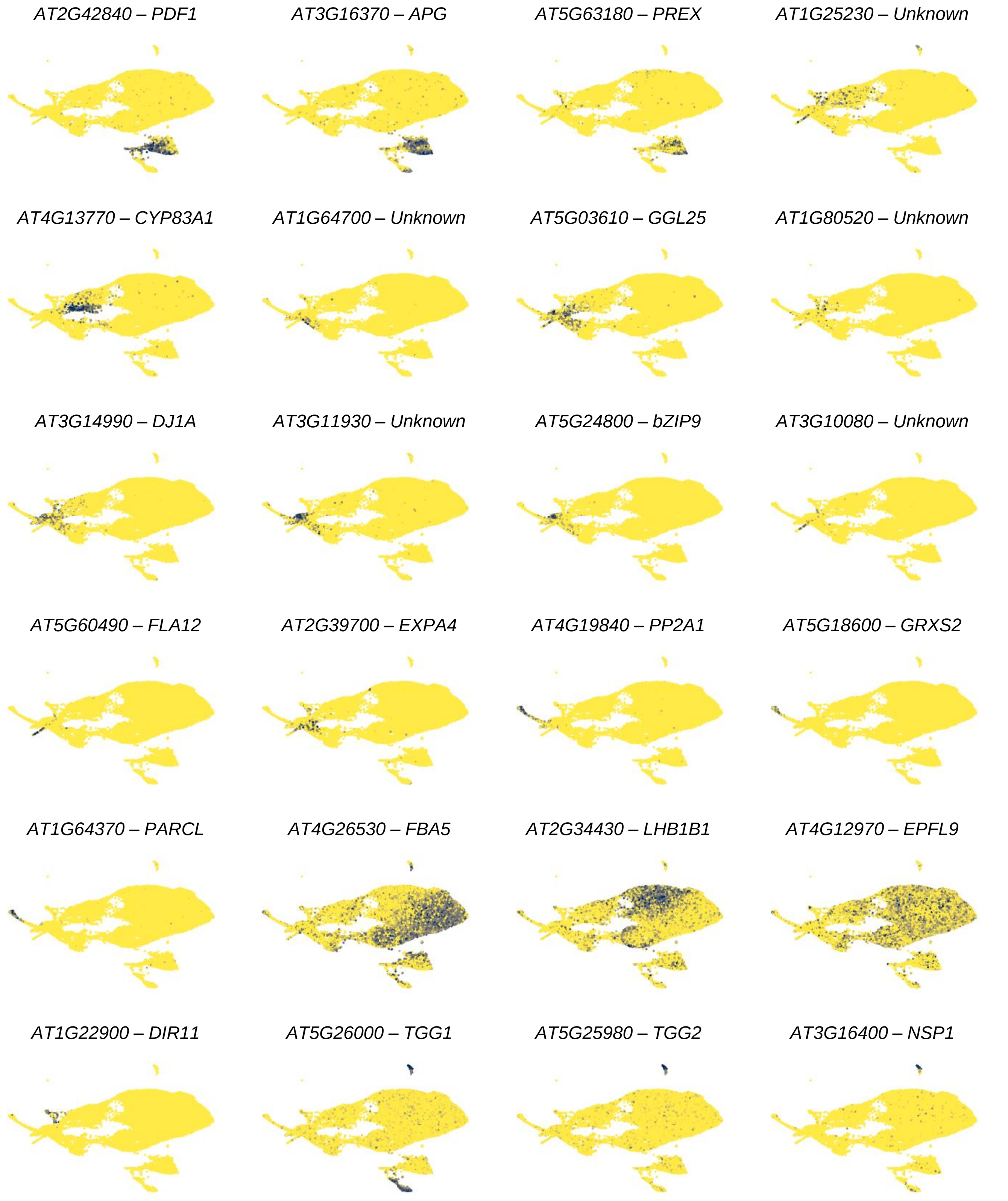

Supp. Figure 15. Expression of additional tissue-specific marker genes. UMAP plots depicting the normalized expression (blue = highest expression) of additional marker genes for each tissue of the Arabidopsis leaf: epidermis (*PDF1*, *APG*), pavement cell (PREX), bundle sheath (AT1G25230, *CYP83A1*), vasculature (AT1G46700, *GGL25*, AT1G80520 and *DJ1A*), phloem parenchyma (AT3G11930, *bZIP9*), xylem (AT3G10080, FLA12), cambium (EXPA4), phloem companion cell (*PP2A1*, *GRXS2*, *PARCL*), mesophyll (*FBA5*, *LHB1B1* and *EPFL9*), hydathode (*DIR11*), myrosin idioblast (*TGG1*, *TGG2* and *NSP1*). UMAP = Uniform Manifold Approximation and Projection

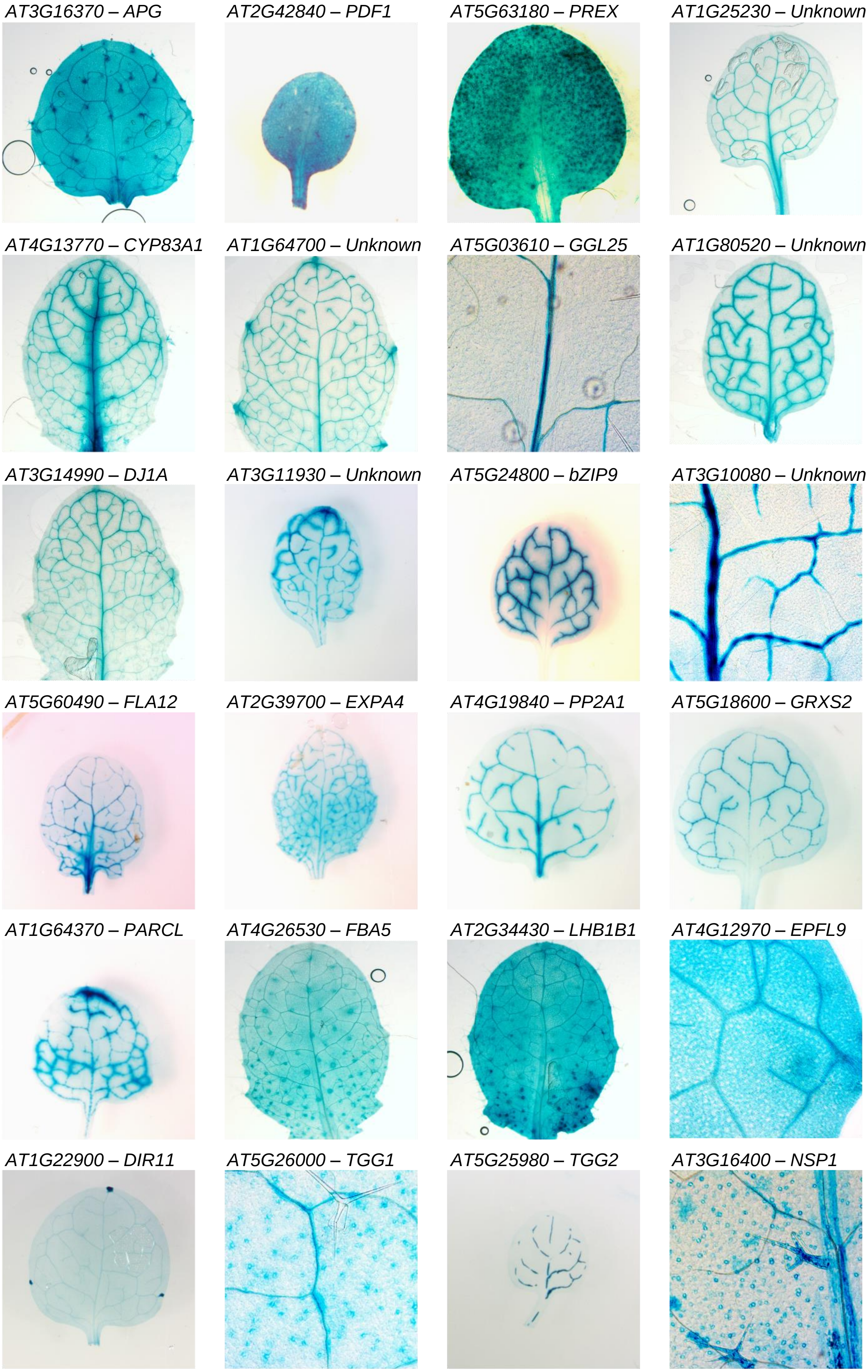

Supp. Figure 16. GUS staining images for the reporter lines of each Arabidopsis leaf tissue. Top-view images of true leaves are shown. GUS = β-glucuronidase.

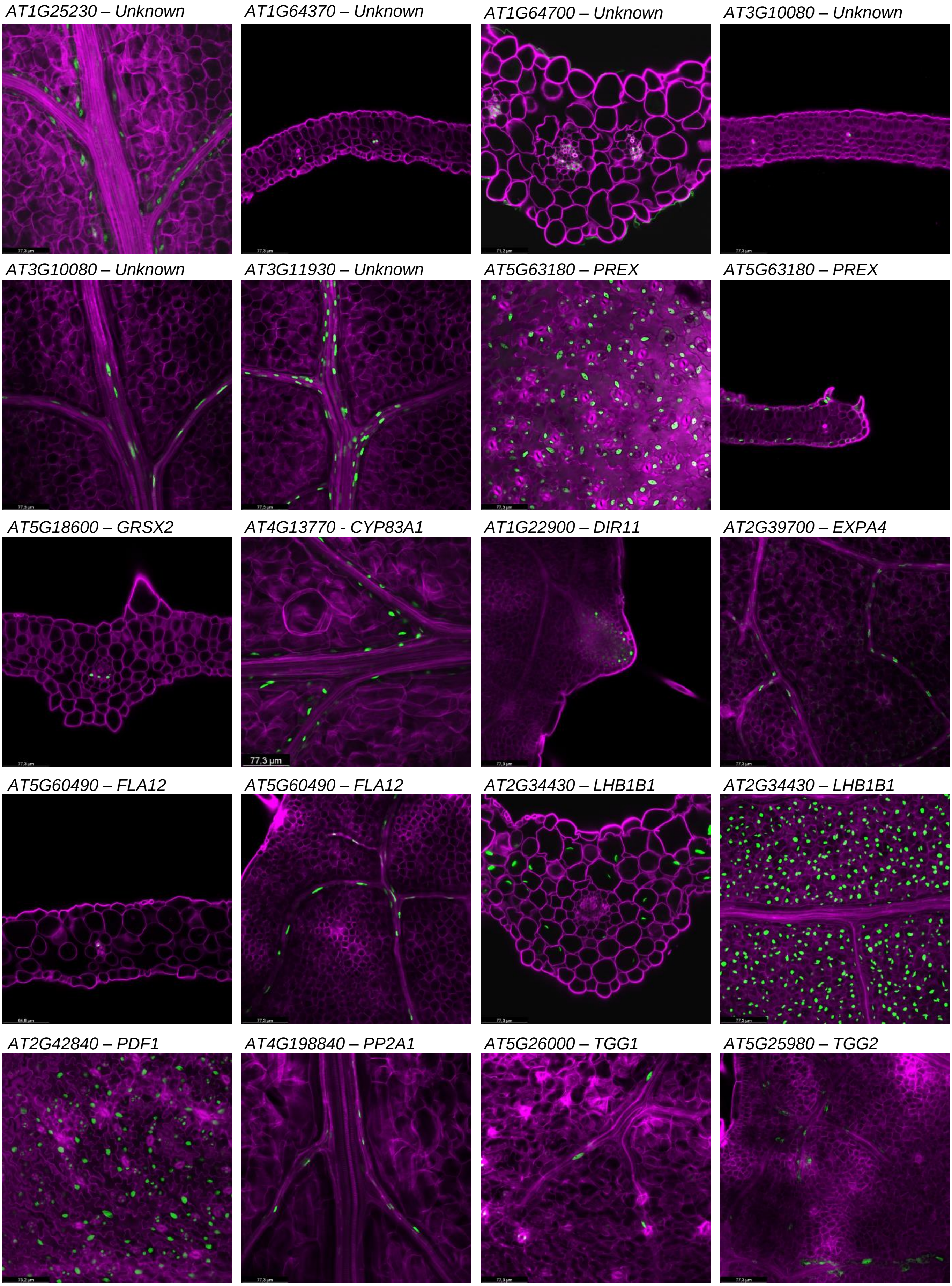

Supp. Figure 17. Additional confocal microscopy images for the reporter lines of each Arabidopsis leaf tissue. Top-view images and leaf sections are shown.

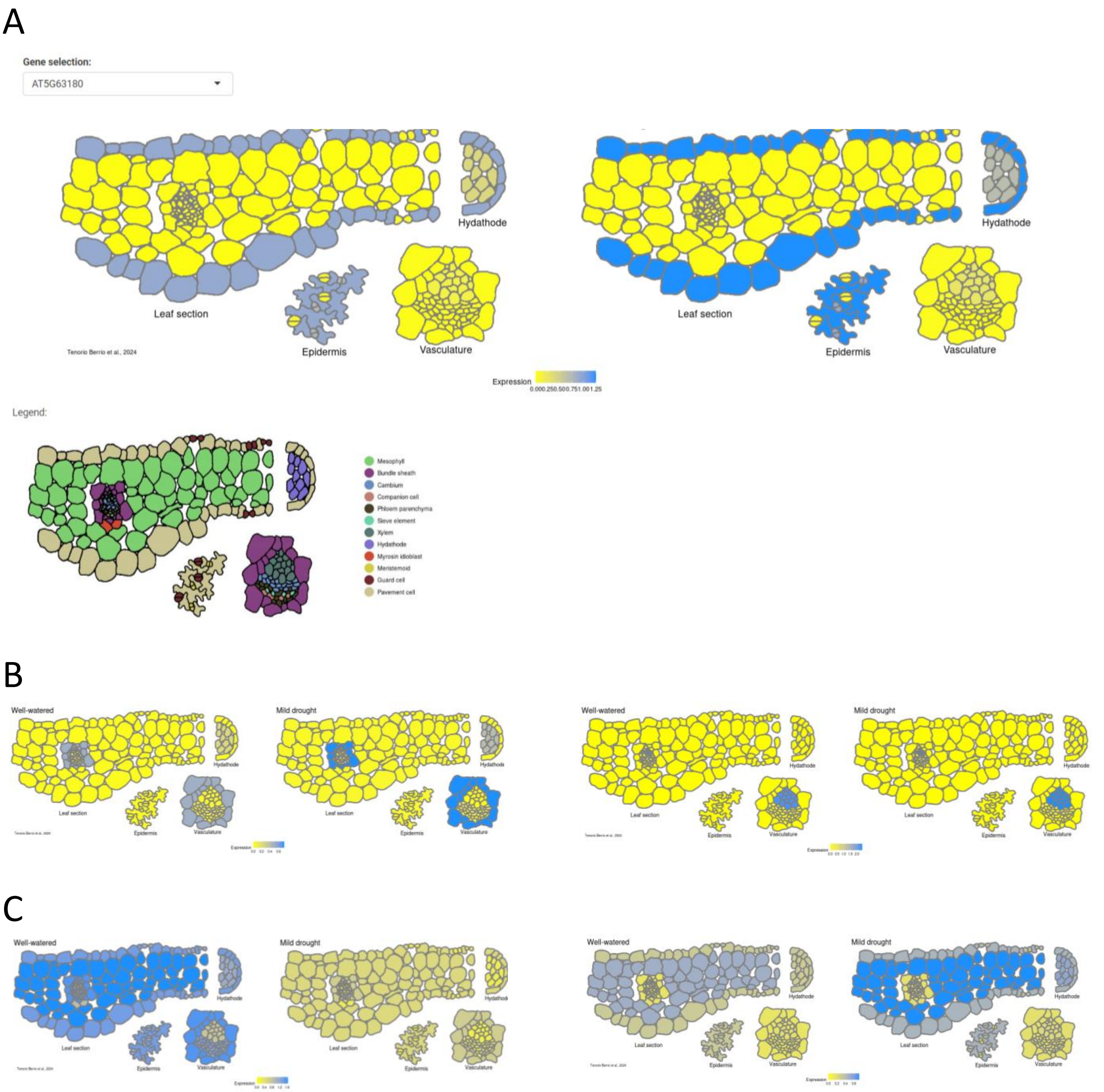

Supp. Figure 18. Tool for single-cell data visualization in an Arabidopsis leaf section. (A) Screenshot of the application (available through https://single-cell.be/plant/leaf-drought > TREX) for visualizing the expression of any gene of interest in a schematic section of the leaf and in the epidermis. The depicted gene is the pavement cell marker *PREX* (AT5G63180). **(B)** Additional examples of tissue-specific markers *SULTR* (left) and *FLA12* (right). **(C)** Additional examples of drought-responsive genes *CSD2* (left) and *AFL1* (right).

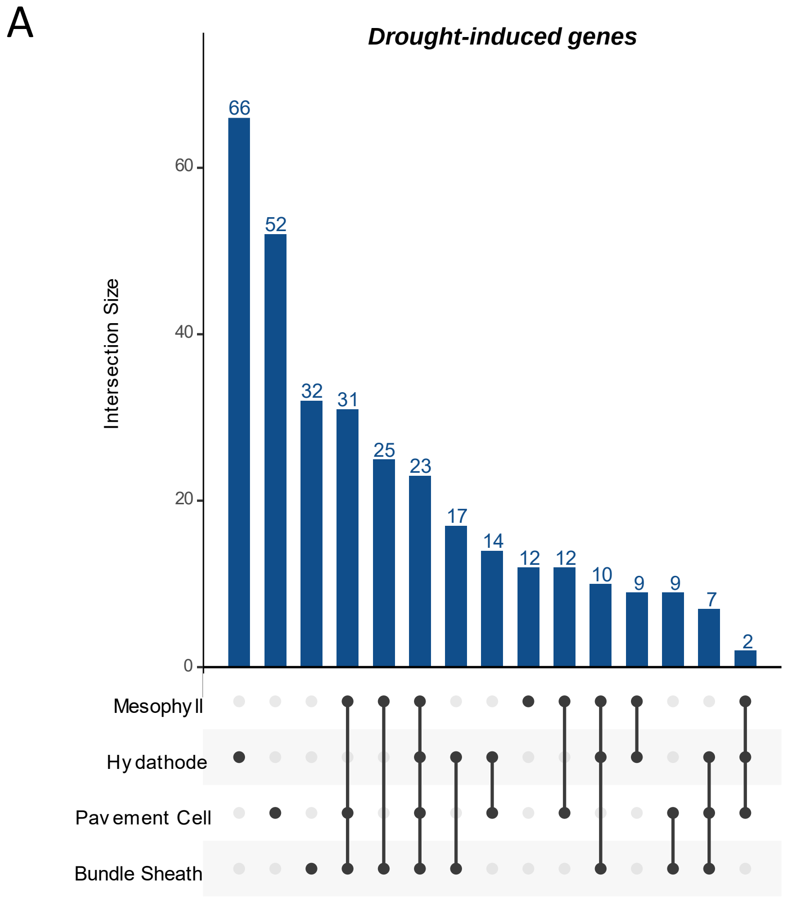

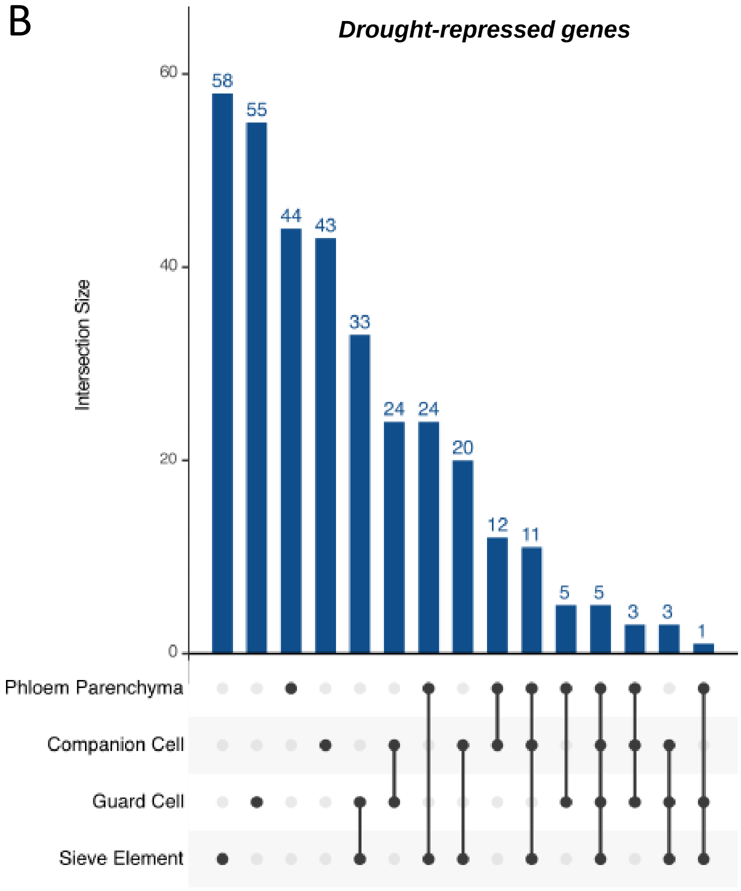

Supp. Figure 19. Mild drought responses shared between Arabidopsis leaf tissues. Upset plot visualization of the number of common genes responding to drought among top responding tissues according to Figure 4B, depicted in the lower panel, between the genes induced **(A)** or repressed **(B)** upon mild drought. Bar plots in the upper panel depict the sizes of the gene sets in the intersections indicated in the lower panel.

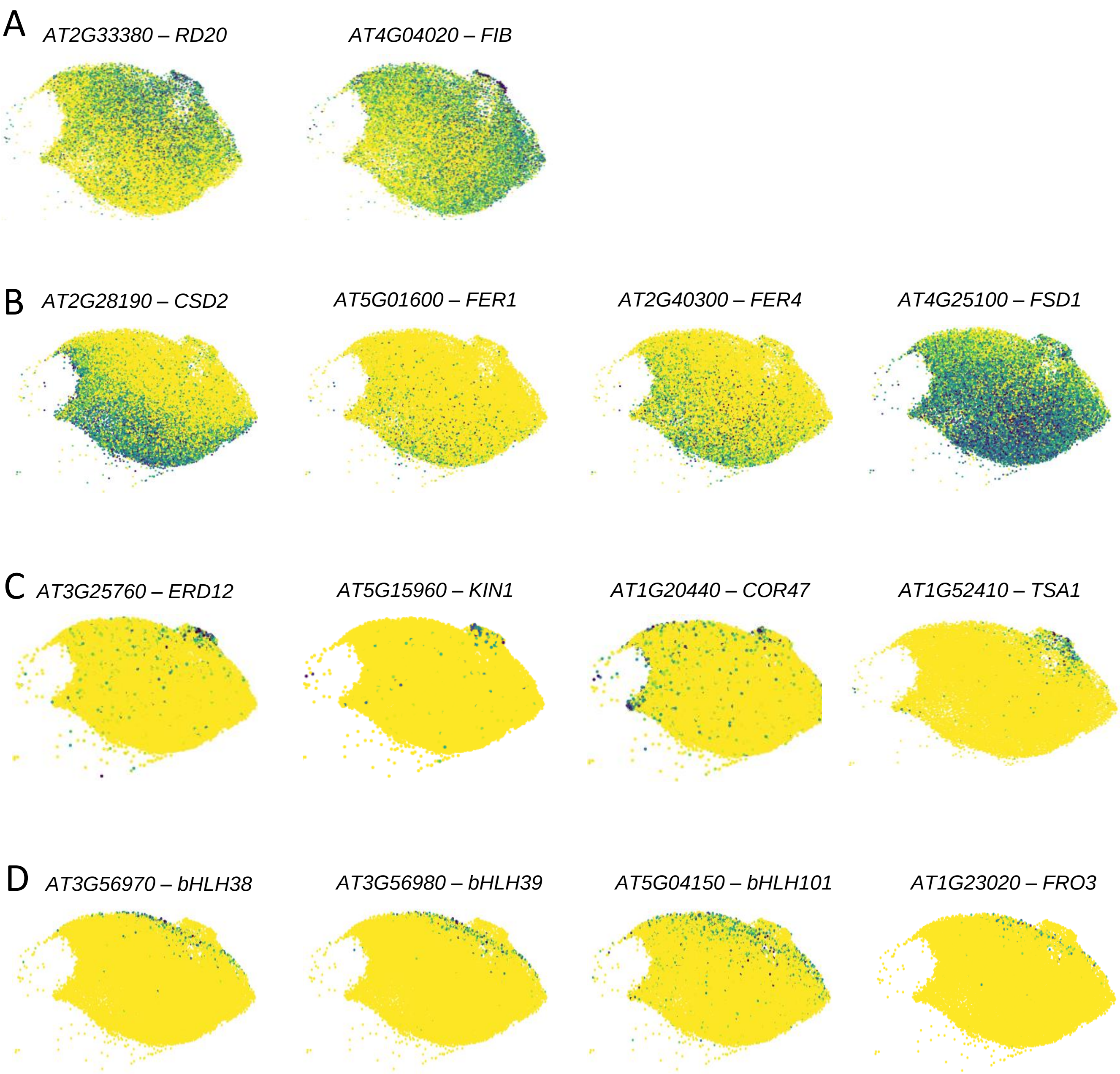

Supp. Figure 20. Expression of drought-related genes in the Arabidopsis mesophyll tissue. (A) UMAP plots depicting the normalized expression of the drought-induced genes *RD20* and *FIB* following the drought stress gradient. **(B)** UMAP plots depicting the normalized expression of *CSD2* and iron-related genes with decreasing expression along the drought stress gradient (described in Figure 2G). **(C)** UMAP plots depicting the normalized expression of genes expressed in the very tip of the drought gradient. **(D)** UMAP plots depicting the normalized expression of genes related to iron starvation. UMAP = Uniform Manifold Approximation and Projection

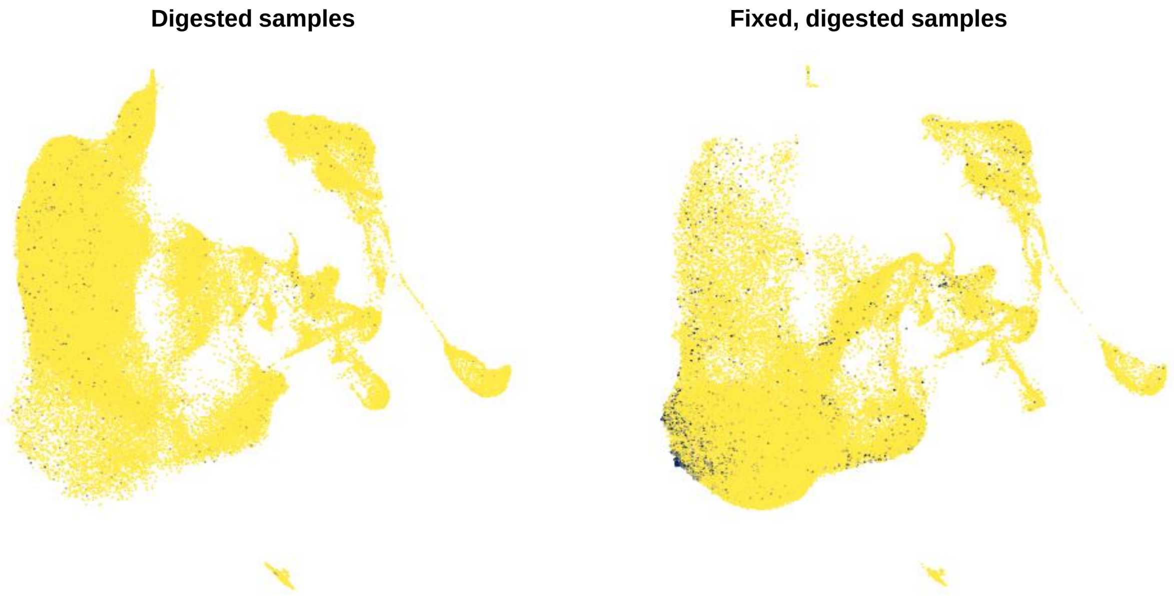

Supp. Figure 21. Expression of *BGLU18* in the combined Arabidopsis leaf scRNA-seq dataset. UMAP plots depicting the normalized expression of *BGLU18* in the combined dataset, split by cell isolation method. UMAP = Uniform Manifold Approximation and Projection

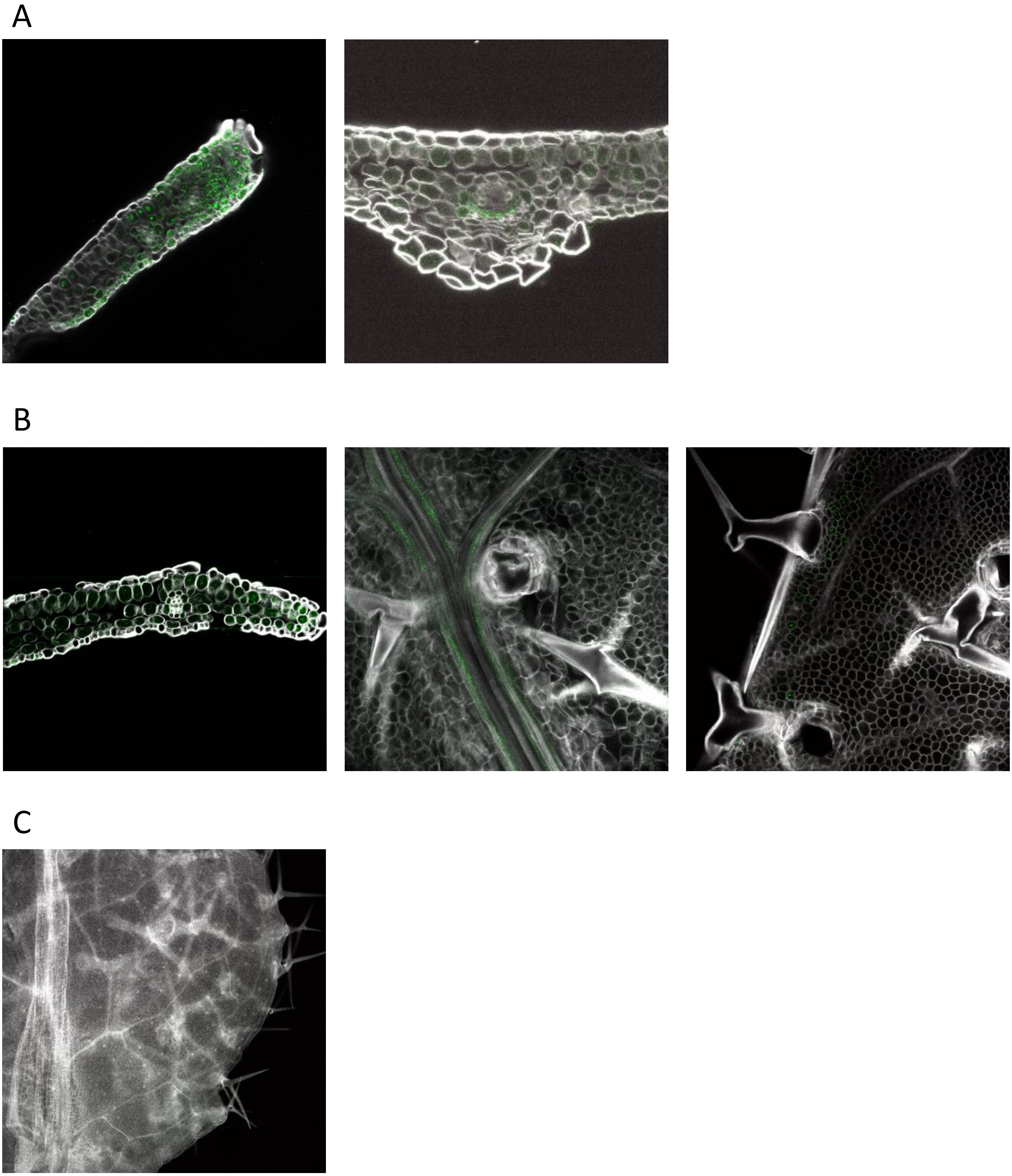

Supp. Figure 22. Transcript visualization of canonical drought responses in Arabidopsis leaves. Confocal microscopy of whole-mount fluorescence *in situ* hybridization (HCR-FISH) targeting the *BGLU18* **(A)**, *TSA1* **(B)** transcripts and a negative control **(C)** upon mild drought conditions.

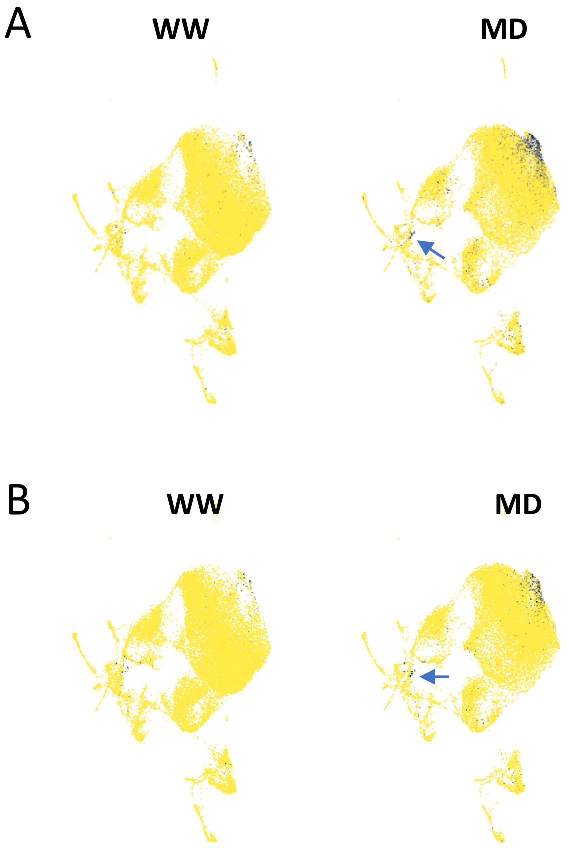

Supp. Figure 23. Expression of *BGLU18* (A) and *TSA1* (B) in the Arabidopsis leaf single-cell dataset of the Fixed-Digested samples. UMAP plots depicting the normalized expression of both genes in the curated dataset, split by well-watered or mild drought treatment. The arrow highlights the expression in a small vascular population of which the precise identity is yet to be determined. UMAP = Uniform Manifold Approximation and Projection, WW = well-watered, MD = mild drought

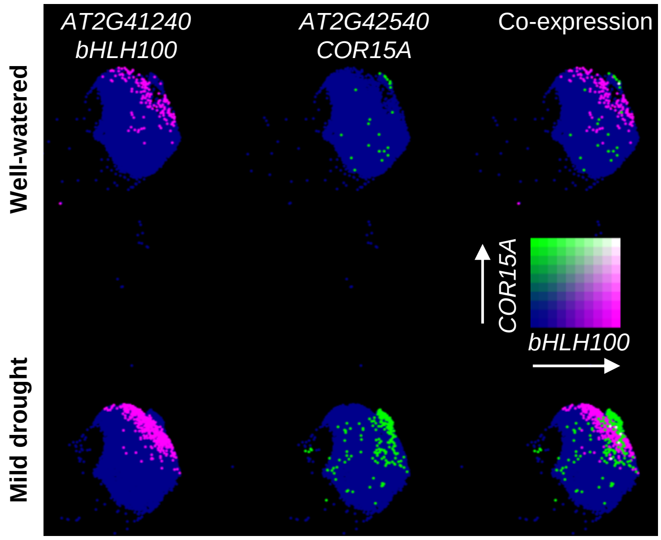

Supp. Figure 24. Co-expression of dual drought responses in the Arabidopsis mesophyll. UMAP plot depicting the expression of *bHLH100* and *COR15A* in the well-watered and mild drought samples in the mesophyll. UMAP = Uniform Manifold Approximation and Projection

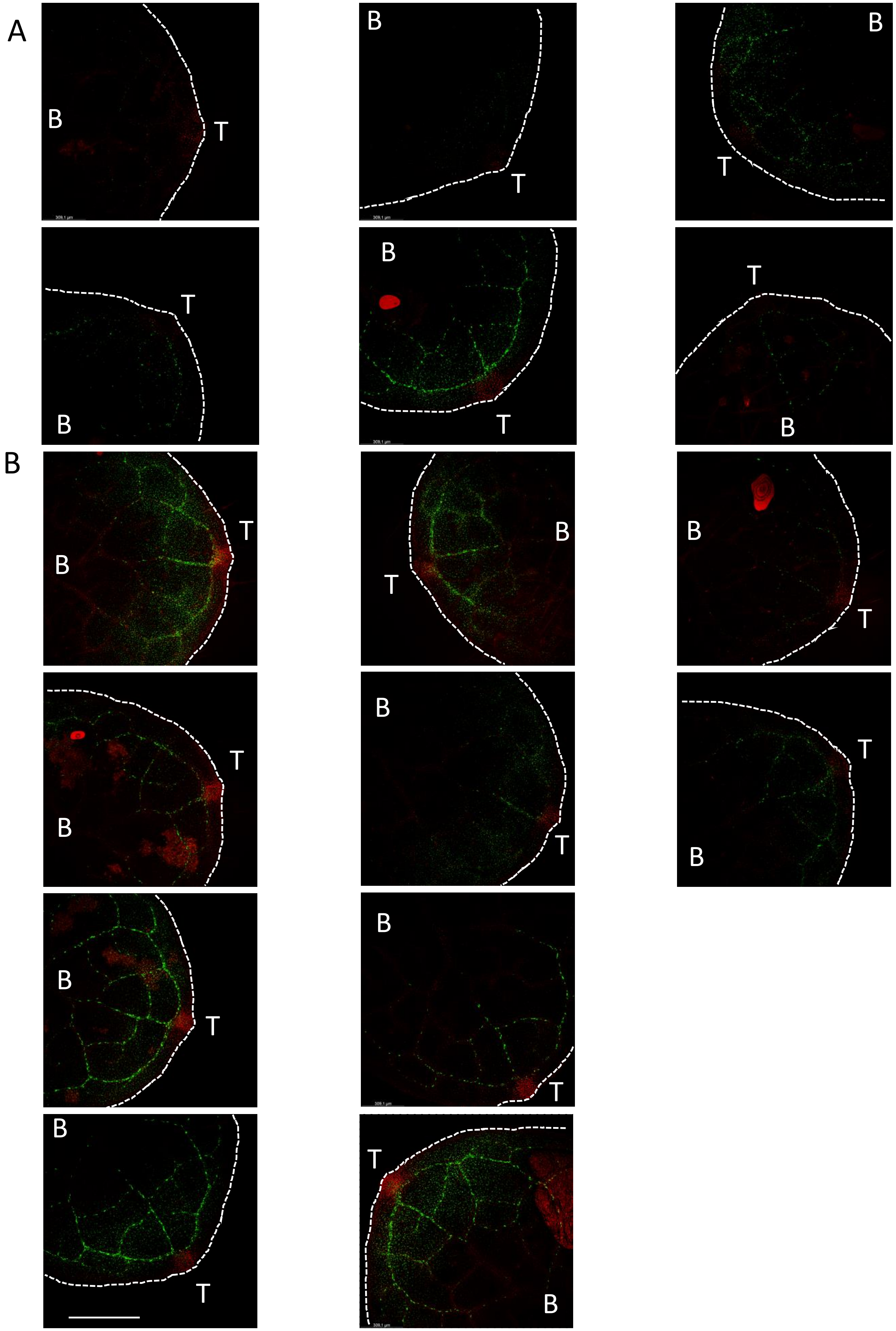

Supp. Figure 25. Microscopic images used for image analysis of the dual drought response. Top-view images of p*bHLH100*::nls-GFP x p*COR15A*::nls-mCherry leaves upon well-watered **(A)** or mild drought **(B)** conditions. Dashed lines delineate the margins of the leaf. T and B mark the position of the tip (T) and the base (B) of the Arabidopsis leaf.

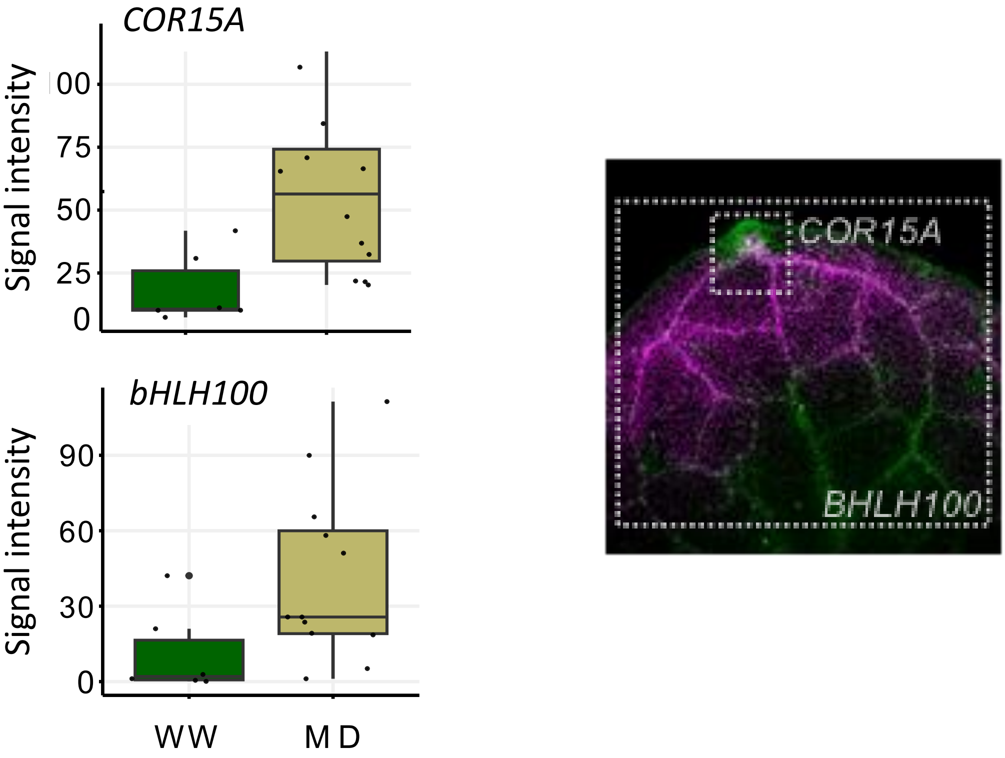

Supp. Figure 26. Quantification of the dual fluorescent signal. Signal intensity quantification of the images shown in Supp. Figure 25, in the zones indicated in the picture, from leaves of Arabidopsis plants grown under well-watered (WW) and mild drought (MD) conditions. In the boxplot, dots represent individual measurements, the horizontal line represents the average.

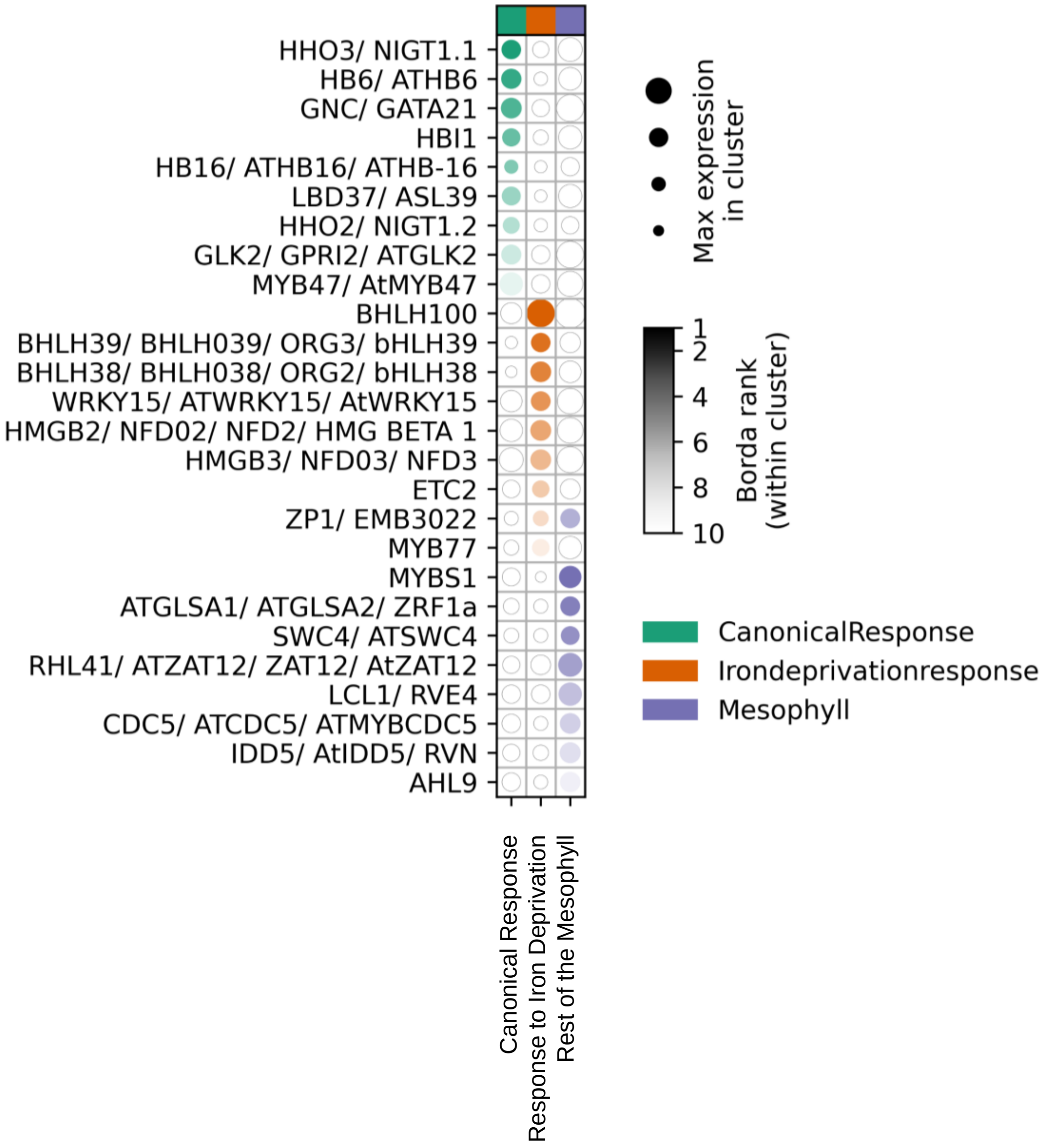

Supp. Figure 27. Regulatory map (regmap) output of the MINI-EX analysis. The top-10 regulators per cluster of the Arabidopsis mesophyll are shown. The full output of the analysis is presented in Supplemental Table 9. MINI-EX = Motif-Informed Network Inference based on single-cell EXpression data

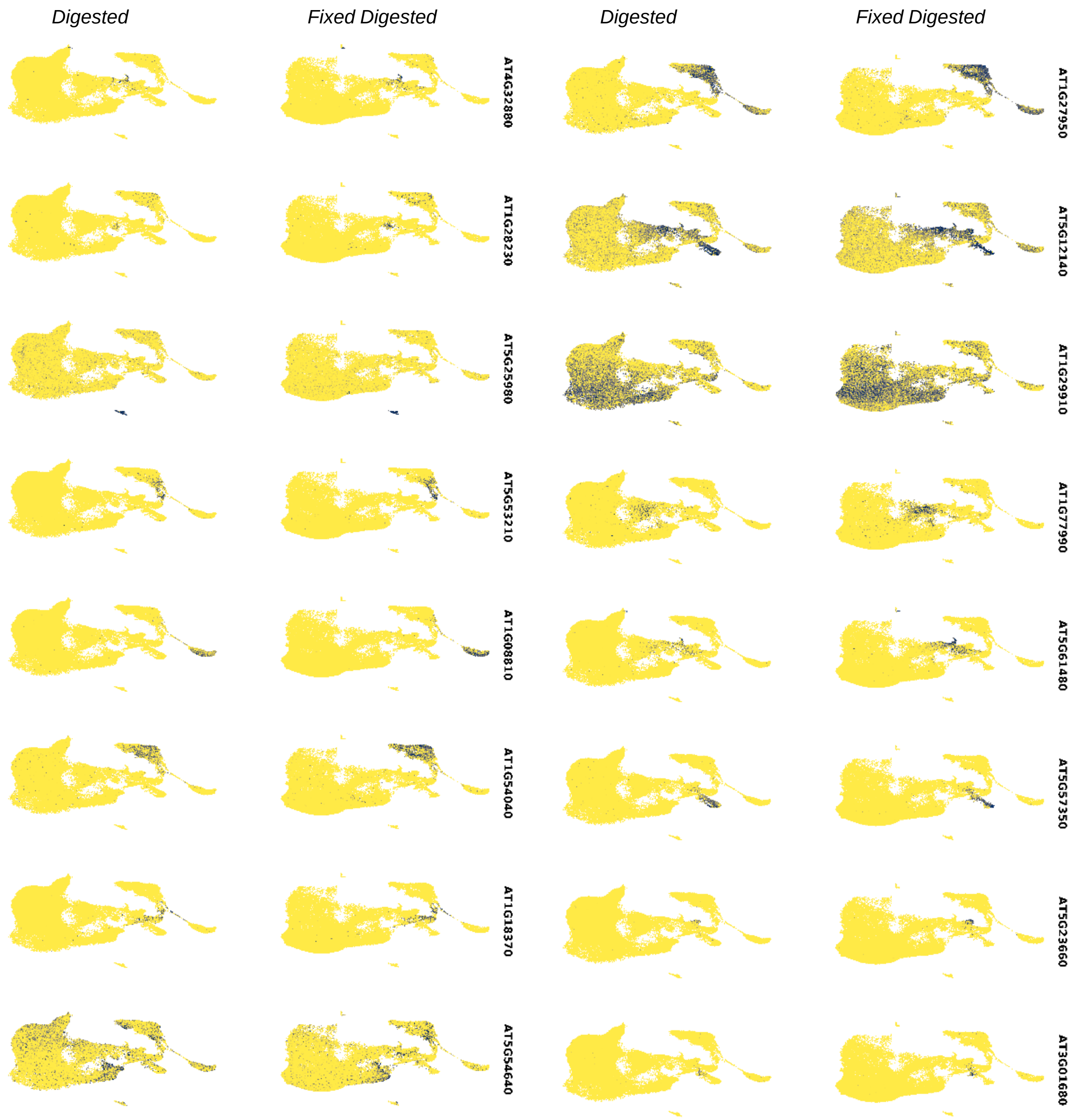

Supp. Figure 28. Expression of tissue-specific marker genes in the combined Arabidopsis leaf scRNA-seq dataset. UMAP plots depicting the normalized expression of the marker genes displayed in Figure 2H, split by sample. UMAP = Uniform Manifold Approximation and Projection
