# Supplementary material for: Dual and spatially resolved drought responses in the Arabidopsis leaf mesophyll revealed by single‐cell transcriptomics
Source: New Phytol. 2025 Mar 3;246(3):840–58. doi: 10.1111/nph.20446 (PMC11982798; doi:10.1111/nph.20446)
Supplement: Supplementary file 4 — Table S3 Information and metadata parameters of the scRNA‐seq dataset. [file NPH-246-840-s008.pdf]

New Phytologist Supporting Information

Article title: Dual and spatially resolved drought responses in the Arabidopsis leaf mesophyll revealed by single-cell transcriptomics

Authors: Rubén Tenorio Berrío, Eline Verhelst, Thomas Eekhout, Carolin Grones, Lieven De Veylder, Bert De Rybel, Marieke Dubois

Article acceptance date: 13 January 2025

Supplemental Table 3: Necessary reported information to allow evaluation and repetition of a plant single cell/nucleus experiment

	Details	Experimental information
**Biological material**	Species	*Arabidopsis thaliana*
	Accession	Col-0
	Genotype	Wild type
	Tissue type	Third true leaf, at 14 days after stratification
	Detailed growth conditions	Arabidopsis Col-0 was grown under a long-day regime (intensity 110–120 µmol m−2s−1) at 21 °C on the WIWAM platform (http://www.wiwam.be). Five seedlings were grown per pot in order to obtain 80 seedlings per conditions, per biological replicate. The seeds were sown in 85±0.5 g of Saniflor compost (Van Isreal N.V., Geraardsbergen, Belgium) with an average absolute water content of 70%. Plants were watered daily until 8 days after stratification (DAS) with a well-watered regime of soil relative water content (RWC) 69% (2.2 g_{water}/g_{soil}). At 9 DAS, half of the pots (randomized) were not watered (MD) until harvest (14 DAS) while the other half kept the well-watered (WW) regime. The second biological replicate was performed ~6 months after the first one.
	Harvest conditions	Per sample, ~80 leaves were harvested with a tweezer and pooled for sample preparation.
**Sample preparation**	Isolation protocol	None (full leaves were used)
	Tissue dissection	Protoplasting protocol was adapted from (Ryu et al., 2019). Briefly, the third leaf of 80 plants per sample was harvested, chopped and incubated in 1 ml cell wall digestion solution (0.4 M mannitol, 10 mM $CaCl_2$, 20 mM MES, 20 mM KCl, 2% (wt/vol) cellulase R-10 and 0.5% (wt/vol) macero enzyme R-10) and subsequently transferred to a 70-µm cell strainer placed in a 6-well plate. Extra cell wall digestion solution was added up to 5 ml. For the samples in which transcription had to be inhibited, Actinomycin D (ActD) was added to a concentration of 50 µM. Cell wall digestion was done by constantly shaking for 75 min at room temperature. After a 7-min centrifugation at 100 g, the pellet was resuspended in 1 ml washing solution (0.4 M mannitol, 10 mM $CaCl_2$, 20 mM MES and 20 mM KCl).
	Fixation	Transcription was inhibited with 50 µM Actinomycin D, applied to half of the samples from each growth condition.
	Cell/nuclei enrichment	LeviCell magnetic sorter
	Total sample preparation time	~2h
	Estimated cell/nuclei number loaded	25000
	Instrument/Method/Kit	BD Rhapsody HT

	Cell viability test	-
**Libraries**	Library construction	BD Rhapsody WTA 23-24117(02)
	Amplification method	13 cycles were used for cDNA amplification and 8 for index PCR.
	End bias	3’
**Sequence results**	Instrument/method	NextSeq2000/<br>NovaSeq6000
	Library layout/paired-end	Paired-end
	N° sequenced reads	At least 25k/cell
**Raw data**	Reference genome	TAIR10
	Annotation version	Release 40
	Mapping method (incl. software, customized settings)	STAR (BD Rhapsody pipeline)
	Mapping efficiency	> 78%
	Sequencing saturation	> 72%
	Estimation of ambient RNA	-
	Imputation method and settings	Fraction reads in cells > 80%
**Processed data**	N° captured cells/nuclei	WW_rep1: 553.211 (No filter), 36.140 (350 min genes filter)<br>MD_rep1: 527.586 (No filter), 25.509 (350 min genes filter)<br>WW_F_rep1:590.774 (No filter), 36.205 (350 min genes filter)<br>MD_F_rep1: 380,.757 (No filter), 17.498 (350 min genes filter)<br>WW_rep2: 245.673 (No filter), 90.332 (350 min genes filter)<br>MD_rep2: 224.141 (No filter), 71.286 (350 min genes filter)<br>WW_F_rep2: 287.720 (No filter), 54.777 (350 min genes filter)<br>MD_F_rep2: 217.425 (No filter), 53.076 (350 min genes filter)
	N° high quality cells/nuclei	WW_rep1: 20.330<br>MD_rep1: 16.273<br>WW_F_rep1: 22.502<br>MD_F_rep1: 11.473<br>WW_rep2: 27.742<br>MD_rep2: 21.544<br>WW_F_rep2: 16.139<br>MD_F_rep2: 16.790
	Filter criteria: % mitochondrial reads/cell or nucleus	-
	Filter criteria: % chloroplast reads/cell or nucleus	-

	Filter criteria: Minimum N° UMI/cell or nucleus	1250 UMI and 5 genes per cell
	N° total detected transcripts	26,150
	Doublet rate	Doublet score calculated identified with scds (Bais and Kosta, 2020), (v.1.2.0).
	Replicate comparisons	Batch correction was performed for the factors Replicate, because both independent replicates were performed with ~6 months time interval, leading to large transcriptional variation between both repeats. In addition, in the combined dataset (D + FD), batch correction batch correction was also applied for the fixation treatment (Replicate + Treatment), due to the transcriptomic impact of fixation.
	Batch correction method for merging (incl. reasoning for batch correction)	After trying several batch correction methods, harmony batch correction was the preferred option. Batch correction was performed for the factors Replicate and Treatment.
	Additional processing	Having established the added value of applying the transcription inhibitor ActD, we only proceeded with the ActD-treated samples. From the ActD-treated samples, we removed the cells that still showed a high response to cell wall digestion, as if they escaped the ActD treatment. This was performed by scoring the cells on the expression of cell wall digestion-induced genes (see manuscript).
**Validation**	Method of automatic annotation of clusters	-
	Method of manual annotation (markers, gene function info)	To predict the annotation of the clusters of the final dataset obtained from fixed samples, we analyzed the expression of previously reported tissue-specific genes (Tenorio Berrio et al., 2022, Kim et al., 2021).
	Verification in planta (e.g. Number of markers used for validation)	We generated reporter lines for 25 genes with tissue-specific expression for most of the predicted cell types, by fusing the promoter region to the nuclear-localized Green Fluorescent Protein (GFP) and β-glucuronidase (GUS) genes. After verifying their expected expression pattern by GUS staining, we selected one reporter line per tissue for in depth validation by confocal microscopy on leaf sections. See manuscript for the full list of reporter lines used.
**Data availability**	Analysis scripts & codes (GitHub)	https://github.com/mariekedubois/TenorioBerrio_2024
	Excel Tables DEG for each cluster	See Supplemental Material from the manuscript.
	Objects/count matrix in repository (which one, where?)	Raw and processed data of the scRNA-seq experiments can be accessed at NCBI with respective GEO number GSE273033.
	On-line tool/browser URL	www.single-cell.be/plant/leaf-drought
	Cell-level metadata table	See Supplemental Material from the manuscript.

**Additional**	additional comments from the authors	-
